# Femto-joule threshold reconfigurable all-optical nonlinear activators for picosecond pulsed optical neural networks

**DOI:** 10.1038/s41377-025-02175-4

**Published:** 2026-02-27

**Authors:** Ruizhe Liu, Zijia Wang, Chuyu Zhong, Yan Chen, Boshu Sun, Jialing Jian, Hui Ma, Dawei Gao, Jianyi Yang, Lan Li, Kaihui Liu, Xiaoyong Hu, Hongtao Lin

**Affiliations:** 1 The State Key Lab of Brain-Machine Intelligence, Key Laboratory of Micro-Nano Electronics and Smart System of Zhejiang Province, College of Information Science and Electronic Engineering, Zhejiang University, Hangzhou, 310027 China; 2 Shenzhen Technology University, College of Integrated Circuits and Optoelectronic Chips, Shenzhen, 518118 China; 3 Zhejiang Key Laboratory of 3D Micro/Nano Fabrication and Characterization, Westlake Institute for Optoelectronics, Fuyang, Hangzhou, Zhejiang 311421 China; 4 Zhejiang Key Laboratory of 3D Micro/Nano Fabrication and Characterization, School of Engineering, Westlake University, Hangzhou, Zhejiang 310030 China; 5 College of Integrated Circuits, Zhejiang University, Hangzhou, 310027 China; 6 Institute of Advanced Technology, Westlake Institute for Advanced Study, Hangzhou, Zhejiang 310024 China; 7 State Key Laboratory for Mesoscopic Physics, Frontiers Science Center for Nano-optoelectronics, School of Physics, Peking University, Beijing, 100871 China

**Keywords:** Silicon photonics, Integrated optics, Optoelectronic devices and components, Optical properties and devices

## Abstract

Achieving optical computing with thousands of tera-operations per second per watt per square millimeter (TOPs/W/mm^2^) is the key to surpassing electrical computing. This realization requires a breakthrough in the design of a new optical computing architecture and nonlinear activation functions. By leveraging the Kerr effect of silicon and the saturable absorption of graphene, we designed an all-optical nonlinear activator based on a graphene-silicon integrated photonic crystal cavity. The ultralow-threshold, high-speed, compact, and reconfigurable all-optical nonlinear activator could achieve a saturable absorption energy threshold of 4 fJ and a response time of 1.05 ps, a reconfigurable nonlinear activation threshold of 30 fJ and a response time of 4 ps, and an ultrasmall size of 15 μm × 10 μm. This device provides foundation blocks for the picosecond pulsed optical neural network chip to achieve 10^6^ TOPs/W/mm^2^ level optical computing.

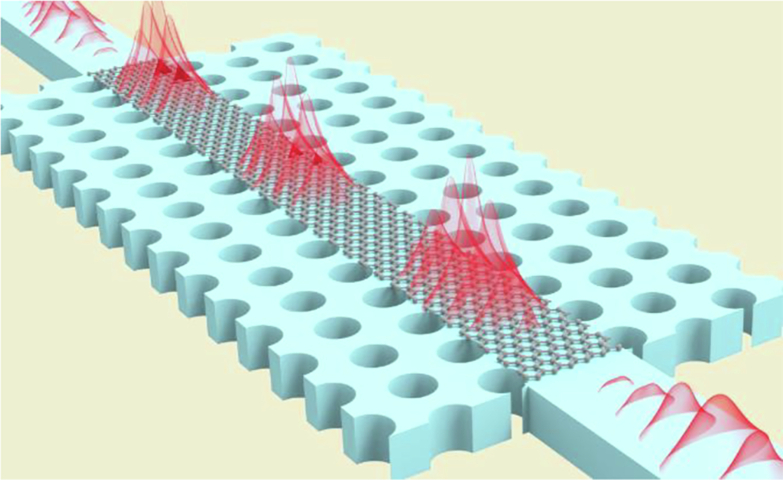

## Abstract (web summary)

We developed a graphene-silicon integrated photonic crystal cavity-based all-optical activator (ultralow threshold, fast, compact, reconfigurable) enabling picosecond optical neural network chips with high-efficiency computing.

## Introduction

Neural networks, inspired by the information processing mechanisms of the biological nervous system, represent powerful machine learning models^1^. However, traditional electronic-based artificial neural networks face bottlenecks in terms of computational speed and energy consumption^2^, which are limited to within 1 TOPs/W/mm^2^. Compared with neural networks in traditional von Neumann architecture electronic computers^3^, optical neural networks (ONNs) leverage the unique advantages of photons^4^, such as wide bandwidth, low power consumption^5^, high parallelism^6^, and high speed, enabling efficient logical calculations^7^ and matrix operations^8^. This opens up broad prospects for applications in artificial intelligence^9,10^, including image recognition^11,12^, audio classification^13^, and phase transition system analysis^14^ with the potential for ultrafast processing speed and lower power consumption^15^.

ONNs, such as optical diffraction-based neural networks^11,16^ or optical interference-based neural networks^5,13^, simulate the operations of biological synapses and neurons. It involves two main computational processes: (a) linear weighting operations and (b) nonlinear activation^17^. The power consumption for linear weighting can be minimized to near zero once phase-change materials are introduced^18,19^ to achieve nonvolatile devices^20^. Nonlinear activation functions (NAFs) play a crucial role in neural networks^21^ as they introduce nonlinear transformations into the output of neurons. This mechanism allows for the development of complex representations in the network while also preventing issues such as gradient vanishing or explosion and enables the network to automatically learn key features from the data^4^.

The mechanisms of existing NAF devices can be categorized into optoelectronic and all-optical strategies. The optoelectronic NAF devices mostly rely on opto-electro-optic conversion^22,23^ or external electrical circuits to excite nonlinearity of materials such as indium tin oxide-graphene heterojunctions^24^, MoS_2_ opto-resistive RAM switches^25^ and graphene‒silicon heterostructures^26^. However, these devices often suffer from a low density of integration, high power consumption, or slow response speeds because of electro-optical conversion. Current all-optical nonlinear activators (ANAs) are based on the nonlinear characteristics of materials, such as stimulated Brillouin scattering^27,28^, electromagnetically induced transparency^14^, free carrier absorption^29,30^, saturable absorption^31–36^, second harmonic generation and its inverse process^37^, cross-phase modulation^38^, self-phase modulation^39^, exciton–polariton^40–42^, phase change effect^18,43^, and diffractively coupled vertical-cavity surface-emitting lasers^44^. Without the need for optical-electrical conversion driving circuits, these devices could achieve a higher density of integration but still face the challenge of simultaneously achieving low thresholds, high speeds, and reconfigurability, which is important for high-speed, low-power consumption optical computing.

Here, we designed and demonstrated ultrafast reconfigurable ANAs based on a graphene-integrated silicon photonic crystal microcavity with ultralow thresholds and proposed an on-chip picosecond pulsed optical neural network architecture. By introducing the cavity-enhanced Kerr effect, our reconfigurable ANAs can generate multiple types of NAFs, such as linear-like, ReLU-like, and sigmoid-like activation functions for ONNs. Combining the advantages of the ultrafast saturable absorption effect of graphene, this design achieves a saturable absorption energy threshold of 4 fJ and a response time of 1.05 ps, a reconfigurable nonlinear activation threshold of 30 fJ and a response time of 4 ps, which indicates that the state-of-the-art figure of merit surpasses that of other ANAs by more than two orders of magnitude. Compared with linear activation functions, the implementation of our ANAs could also notably enhance the precision of optical neural networks in tasks such as data classification, MNIST, and CIFAR-10 recognition. This nonlinear activator will serve as fundamental building blocks for implementing on-chip picosecond-pulsed optical neural network computing architectures.

## Results

### Silicon-based reconfigurable PhC cavity ANA

**Fig. 1:**
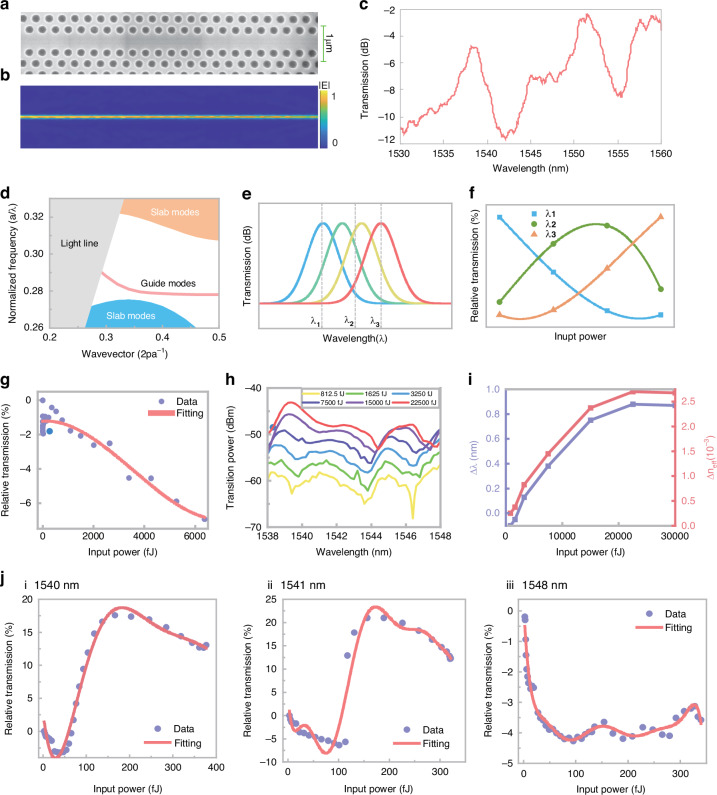
Design, simulation and performance of a silicon reconfigurable PhC cavity ANA. **a** Scanning electron microscope image of the top view of the silicon PhC ANA. **b** Simulated optical mode profile in the PhC device. **c** Normalized transmission spectrum of the PhC cavity ANA. **d** Band structure of the PhC waveguide. The guided mode (red curve) includes a slow-light region. The blue and orange shaded areas indicate the slab modes. **e** Relative transmission curves for three randomly selected wavelengths λ_1_, λ_2_, and λ_3_ (not measured data). **f** Schematic diagram of the redshift of the resonant peak caused by the Kerr effect (not measured data). **g** Nonlinear absorption curve of the PhC cavity device. **h** Transmission spectra of the PhC cavity under different input optical pulse powers. **i** Peak shift at 1539 nm (purple curve) and the change in the effective refractive index (orange curve) at different input optical powers. **j** Reconfiguration activation functions at different wavelengths (ⅰ-ⅲ: 1540 nm, 1541 nm and 1548 nm, respectively)

Owing to the relatively small third-order nonlinear coefficient, exciting third-order nonlinearity on conventional silicon waveguides requires high power, resulting in significant power consumption for device operation^45^. To solve this challenge, a resonant line-defect PhC cavity was designed for reconfigurable ANAs. The device was designed and fabricated on the basis of a standard silicon-on-insulator photonics platform with a two-dimensional periodic circular air hole array and a line defect. A scanning electron microscope image of the device is shown in Fig. 1a. This PhC resonant cavity offers two key advantages: first, by leveraging the slow-light effect^46,47^ of the PhC cavity, the interaction between light and the device is enhanced (Fig. 1b), increasing nonlinear effects and allowing a smaller device footprint. Additionally, we strategically designed the PhC cavity with a relatively weak slow light effect. This approach not only enabled us to reduce the device size but also effectively mitigated the issues of insertion loss and narrow bandwidth, ensuring the overall performance and functionality of the device. Second, through the design of the PhC cavity, light pulses resonate and increase the energy within the device, further enhancing the third-order nonlinear effects^48^. By inducing changes in the effective refractive index of the silicon device through Kerr third-order nonlinearity, which leads to a redshift in the device’s transmission spectrum, as shown in Fig. 1e, multiple types of NAFs can be constructed by selecting different incident light wavelengths on the basis of specific resonant peaks, thereby achieving reconfigurable ANAs (Fig. 1f).

The transmission spectrum of the device was measured via a continuously tunable laser, as shown in Fig. 1c. More details of the measurement system are provided in Section Ⅰ in the Supplementary Information. The nonlinear absorption curve was obtained by measuring the change curve of the device’s transmittance after the input of broad-spectrum femtosecond pulses, as shown in Fig. 1g. Owing to the two-photon absorption effect^49^, the relative transmittance of the device tends to decrease as the input light energy increases. The device exhibited several resonant peaks designed for amplifying third-order nonlinearity (specific design details in Section Ⅱ in the Supplementary Information), with a resonant peak Q factor on the order of hundreds. The femtosecond laser output was coupled into the device through grating coupling, and the output spectra with different input pulse energies are depicted in Fig. 1h. The output spectrum redshifts with increasing input pulse energy, which is attributed to the third-order nonlinear effect in silicon, leading to an increase in the effective refractive index of the silicon cavity and resulting in a redshift of the device’s resonant peaks. This phenomenon could be explained by classical cavity perturbation theory^50^. A simplified formula to calculate the resonant peak shift $\Delta \lambda$ caused by third-order nonlinearity in the microcavity is derived in Section Ⅲ of the Supplementary Information:

1 $$\Delta \lambda =\frac{\Delta {n}_{{eff}}}{{n}_{g}}\cdot {\lambda }_{0}={{n}_{2}}_{{eff}}\cdot Q{P}_{{peak}}\cdot {\lambda }_{0}$$

where $\Delta {n}_{{eff}}$ denotes the waveguide effective index change due to the change in the material index caused by the Kerr effect, ${n}_{g}$ denotes the model group index,$\,{\lambda }_{0}$ represents the probe resonant wavelength, ${{n}_{2}}_{{eff}}$ represents the effective third-order nonlinear coefficient of the waveguide, ${P}_{{peak}}$ represents the pump pulse peak power coupled into the cavity, and the PhC resonant cavity has a quality factor $Q$.

Thus, it can be concluded that the shift of the resonant peak is amplified by the quality factor ($Q$) of the resonant cavity. Figure 1i shows the variation curve of the center wavelength of the resonance peak at 1539–1540 nm with the change in input light power, along with the calculated change in the corresponding effective refractive index $\varDelta {n}_{{eff}}$. These results clearly indicate that upon coupling a femtosecond pulsed laser into the ANA, as predicted earlier, strong third-order nonlinear effects are induced, causing a shift in the device’s resonant peak. The response time is less than 2 ps, as shown in the inset of Fig. 1i.

In addition, the drifts of the resonant peaks make it possible to achieve reconfigurability and programmability of the nonlinear response in the PhC cavity ANA. When a single-wavelength pulse is input, since it is not enhanced by the photonic crystal cavity, the potential two-photon absorption effect is far smaller than the cavity-enhanced Kerr effect. At different wavelengths of the resonant peaks, the trends of the device transmittance change induced by the resonant peak shift vary. In other words, different NAF curves can be generated by changing the wavelength of the incident light. Through the introduction of a filter with a 1 nm 3 dB spectral bandwidth into the saturation absorption measurement setup configuration (Section Ⅰ in the Supplementary Information), the device’s transmittance for picosecond pulses at different wavelengths varied with the input light power. The device, excited by light pulses of less than 500 fJ at other wavelengths, generates distinct activation function curves, with trends in line with the changes in the transmission spectrum shown in Fig. 1j.

Therefore, ANAs with hundreds of femtojoule level thresholds can be reconfigured by taking advantage of the Kerr effects in silicon-based PhC devices. However, the picosecond pulsed optical neural network needs an ANA with a lower threshold for higher-performance optical computing. To further reduce the threshold power of the device, we can approach it from multiple aspects. On the one hand, we can further optimize the design of the PhC cavity, increase its quality factor, enhance the cavity’s ability to concentrate the energy of optical pulses, and thus strengthen the Kerr effect in the cavity. On the other hand, we can optimize the device fabrication process, further reduce device losses, improve energy utilization efficiency, and thereby further lower the activation threshold. In addition, by integrating with graphene materials, we can utilize their excellent optical properties such as saturable absorption to enhance the nonlinear response of the device. We will discuss this in detail in the next section.

### Femto-joule threshold graphene-silicon PhC ANA

**Fig. 2:**
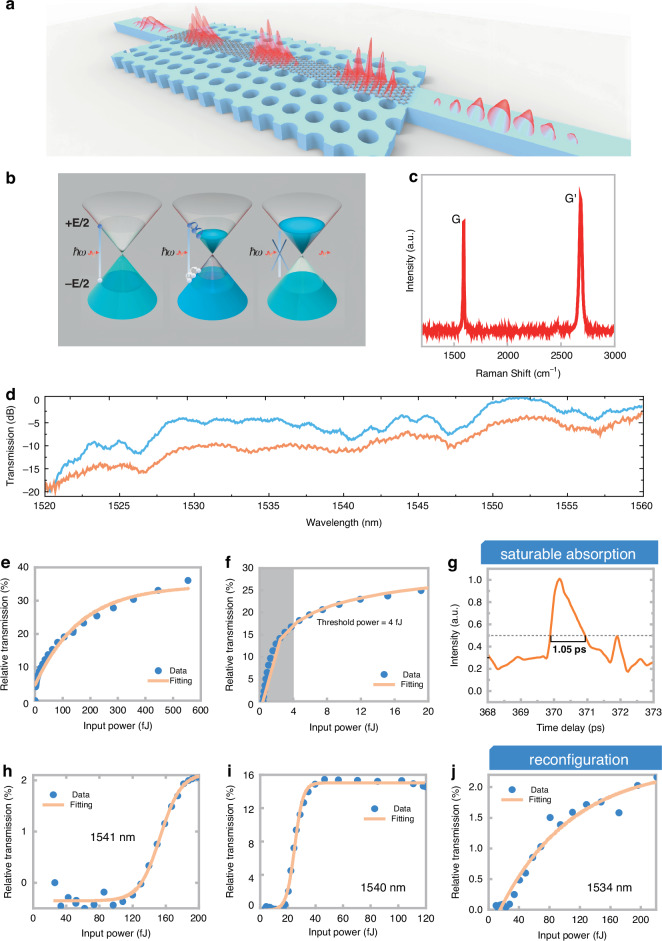
Principle, properties and performance of graphene-silicon PhC cavity ANAs. **a** Three-dimensional schematic of the reconfigurable PhC microcavity ANA. **b** Schematic diagram of the saturable absorption of graphene. **c** Raman spectrum of graphene. **d** Normalized transmission spectra of a PhC cavity ANA without graphene (blue curve) and with graphene (orange curve). **e** Saturable absorption curve of a straight waveguide covered with a length of 15 μm of graphene. **f** Saturable absorption curve of the PhC cavity with graphene, with a threshold power of 4 fJ (50% saturation transmission rate). **g** Change in the transmission of the probe light as a function of its time delay relative to the pump light. The full width at half maximum response time is approximately 1.05 ps. **h–j** Reconfiguration activation functions at different wavelengths

To further reduce the threshold of the ANA, the graphene material was integrated into the silicon PhC cavity (Fig. 2a). As shown in Fig. 2b, owing to the Pauli blocking effect, the optical absorption of graphene gradually decreases with increasing light intensity, and once the intensity exceeds the threshold power, it saturates, with a femtosecond-level response time^51–53^.

Therefore, by leveraging the saturable absorption effect of graphene, we designed a graphene-silicon PhC cavity ANA. Graphene was transferred to the PhC device via a standard wet transfer process^50^ and patterned through electron beam lithography. Figure 2c shows the Raman spectrum of graphene transferred to the sample. The fabrication process flow of our devices and the material properties of the graphene are shown in Section Ⅳ in the Supplementary Information. Figure 2d shows the transmittance spectra of the device before and after graphene transfer. Although the transfer of graphene increases the device’s losses, the resonant peaks are preserved. Owing to the slow-light effect, the interaction between the light pulses and graphene was enhanced^51^, significantly reducing the saturation threshold power of graphene and guaranteeing an ultrafast saturation response time.

To verify the ultralow threshold power and ultrafast response speed of the device combined with graphene, saturable absorption tests and pump-probe tests were performed on a graphene-silicon PhC cavity ANA. The saturable absorption curves are shown in Fig. 2e, f. A comparison between a conventional straight waveguide device covered with 15 μm of graphene (Fig. 2e) and a graphene-silicon PhC cavity ANA (Fig. 2f) reveals an ultralow threshold power of 4 fJ (50% saturation transmittance)^36,54^ due to slow light and cavity-enhanced effects. Compared with the activation threshold of several hundred femtojoules for the silicon photonic crystal cavity ANA device (Fig. 1j), the threshold of the graphene - silicon integrated PhC cavity ANA has been significantly reduced. Additionally, pump-probe measurements were also conducted on the device, as shown in Fig. 2g. The device exhibited increased transmittance after the pump light passed through, returning to its original value within 2 ps, with a full width at half maximum response time of 1.05 ps.

Here, an optical nonlinear switch device with ultralow threshold power and ultrafast response time was realized by combining the graphene saturable absorption effect with the slow light cavity enhancement effect. We survey the current state-of-the-art ANAs in Table 1. Our device has achieved at least four orders of magnitude greater figure of merit than other on-chip ANAs. In addition, by modulating the incident wavelength on the basis of the design of the PhC cavity resonant peaks, multiple different types of NAFs can be achieved.

**Table 1:** Comparison of state-of-the-art ANAs

On-chip ANAs					
Device	Activation energy Threshold	Footprint (μm^2^)	Response time	Reconfigurability	Figure of merit (pJ^–1^ps^–1^)
Si-Gra PhC cavity (Saturable absorption, this work)	4 fJ	~15 × 10	1.05 ps	No	238.1
Si-Gra PhC cavity (Reconfigurable, this work)	30 fJ	~15 × 10	~4 ps	Yes	8.33
Exciton–Polariton^41^	0.6 pJ	N/A	100 ps	No	0.017
PCM on Si^18^	~700 pJ	~100 × 100	0.2 μs	No	7.14 × 10^–9^
Ge-Si PD^30^	~0.27 pJ	~30 × 8	50 ps	No	0.074
Gra modulator^34^	~100 fJ	~40 × 10	<90 ps	No	0.11
Silicon and metal double slots with graphene^36^	0.51 pJ	~20 × 5	100 ps	No	0.02
PCM on Si MRR^43^(free space excitation)	11.9 pJ	N/A	<1 ns	No	8.4 × 10^–5^
SA modulator^35^	10 pJ	N/A	26 ns	No	3.85 × 10^–6^
Stimulated Brillouin scattering in fiber^28^ (potential for on-chip integration)	1 W	N/A	100 ps	Yes	N/A

‘N/A’ indicates that the result is not reported in the literature and cannot be inferred from the data presented

Taking advantage of silicon Kerr third-order nonlinearity effects, as discussed in the above section, the nonlinear response of the graphene-silicon PhC cavity ANA can be reconfigured. When the input pulse was selected near the wavelengths of 1541 nm, 1540 nm, and 1534 nm, ReLU-type NAF(Threshold: 120 fJ)^18^, sigmoid-type NAF(Threshold: 30 fJ)^55^ and linear-type NAF could be achieved, as shown in Fig. 2h–j (details of the configuration can be found in Section Ⅴ in the Supplementary Information). Overall, a wavelength-modulated reconfigurable high-speed ANA has been achieved. The device can realize various NAFs on the basis of the design of the transmittance spectrum, with response times of less than 4.5 ps for activation functions. Clearly, the reconfigurable ANA can saturate at such low power levels with a picosecond response time, indicating the potential for achieving more energy-efficient all-optical neural networks.

### On-chip picosecond pulsed optical neural network and neural network training

**Fig. 3:**
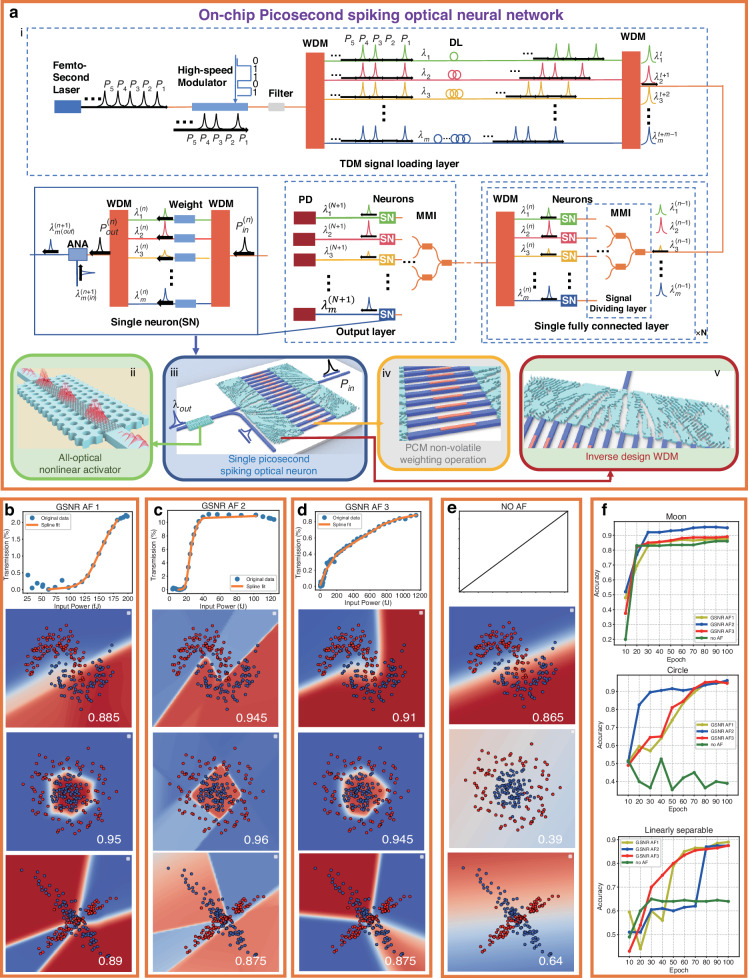
General block diagram of an on-chip picosecond pulsed optical neural network and the performance of graphene/silicon heterojunction nonlinear response activation functions (GSNR AFs) on three binary classification datasets. **a** General block diagram of an on-chip picosecond pulsed optical neural network. **i:** Wavelength division cascaded picosecond pulse optical computing network architecture, which consists of a spatiotemporal misalignment multiplexed signal loading layer, a signal dividing layer, a fully connected layer with picosecond-response nonlinear activation capability, and an output layer. **ii:** A schematic illustration of the reconfigurable PhC microcavity ANA. **iii:** A schematic illustration of a single picosecond pulsed optical neuron, which consists of two inverse design wavelength-division multiplexers (ID-WDMs), m phase change material (PCM) nonvolatile weight operation waveguides, and a reconfigurable photonic crystal (PhC) microcavity ANA. **iv:** A schematic illustration of PCM nonvolatile weight operation waveguides. **v:** A schematic illustration of one-input, m-output ID-WDM. **b–d** ReLU-type, sigmoid-type and linear-type GSNR AFs derived from a graphene-silicon integrated device and their optimal binary classification results on three test sets. **e** Optimal binary classification results on three datasets without any NAFs. **f** Validation classification accuracy results using different activation functions on three datasets

Current on-chip optical computing architectures are based on modulating continuous wave light^56,57^, which has the issue of low power density, making it difficult to activate the material’s nonlinear properties. By using ultrafast pulsed light, it is possible to increase the instantaneous power density without exceeding the material’s thermal damage threshold while effectively stimulating its nonlinear properties. Therefore, pulsed light is highly suitable for realizing all-optical computing architectures. Here, as shown in Fig. 3a, we propose a wavelength division cascaded picosecond pulse optical computing network architecture and analyze the performance requirements of the devices involved.

The entire architecture consists of a spatial-temporal offset-multiplexed signal loading layer signal loading layer, a fully connected layer with picosecond-response nonlinear activation capability, and an output layer, as shown in Fig. 3a.ⅰ. The spatiotemporal misalignment multiplexed signal loading layer includes a high repetition rate picosecond light source, a high-speed broadband modulator, and time-division misalignment units. The picosecond light source with high repetition rate (100 GHz) and narrow pulse width (150 fs) can be implemented through two approaches: off-chip (fiber femtosecond source) or on-chip (mode-locked laser source) solutions (both currently facing significant technical challenges that require further research and development). After being coupled into the on-chip system, the light pulses are encoded by a balanced broadband high-speed modulator (100 GHz bandwidth)^58^. After passing through an on-chip broadband filter, the pulses are split into multiple beams with 1 nm intervals through a wavelength division device (an ID-WDM^59^ as shown in Fig. 3a.ⅴ) and then encoded spatiotemporal misalignment through waveguide delay and combined through an ID-WDM into a waveguide. The spatial thermal noise introduced by fabrication imperfections and the temporal thermal noise caused by thermal fluctuations in WDMs can be compensated for via partially coherent light illumination methods^60–62^.

The fully connected layer with picosecond-response nonlinear activation capability comprises a signal distribution layer, regenerative signal neurons (Fig. 3a.ⅲ) with linear weights (Fig. 3a.ⅳ) and NAFs (Fig. 3a.ⅱ), and a signal bundling layer. The pulses encoded by the last layer are passed through multiple layers of the MMI to distribute the encoded pulse signals to different neurons for processing. Each neuron consists of synapses and activations. The output pulses from the previous layer are sent to an ID-WDM and split into different wavelengths (λ_1_ + λ_2_ + ··· + λ_n_), weighted differently^63^ and combined through an ID-WDM into a waveguide. Next, these pulses pass through an ANA as pump light and are filtered out at the output (the filter is not shown in the architectural diagram). Then, a new single—wavelength pulse (λ_k_, k = 1,2, ···, n. These wavelengths can be the same as those of the previous stage to achieve wavelength multiplexing) is nonlinearly activated and transmitted as the output of a single neuron to the next layer of the network. By cascading and changing the intervals between splitting and wavelength division multiplexing channels and regenerating wavelengths, the scale of the fully connected layer can be arbitrarily changed, achieving matrix compression, pooling, transformation, and other optical computing functional modules. After completing the fully connected operation, the signals are sent to the output layer, which is the signal-fully connected layer that directly connects to high-speed detectors for signal output.

In the picosecond pulsed optical neural network architecture, the multiply-accumulate operations based on phase-change materials exhibit near-zero static power consumption. When the activation energy per computing unit is maintained below 30 fJ, the system demonstrates the potential to achieve computing power density on the order of 10³ TOPS/mm² and computing power energy efficiency density reaching 10⁶ TOPS/W/mm². Consequently, the development of reconfigurable all-optical nonlinear activators (ANAs) featuring ultralow threshold (<30 fJ), picosecond-scale response, and multi-wavelength compatibility will be pivotal for overcoming the power consumption bottleneck in next-generation ultra-high-speed optical computing networks.

To provide an initial assessment of the classification ability of the picosecond pulse optical neural network proposed above, we simplified it into a picosecond pulsed optical fully connected neural network for classification tasks (details of the architecture can be found in Section Ⅵ in the Supplementary Information). We then built a fully connected network based on PyTorch and scikit-learn libraries to simulate its performance. The nonlinear responses generated by our ANAs were fitted into an NAF curve through the linear interpolation method and normalization adjustment (see Section Ⅶ in the Supplementary Information). The NAF curves replaced the classical activation functions in the fully connected network accordingly to solve three kinds of binary classification problems.

Three binary datasets are generated for statistical analysis: concentric circles, crescent moon shapes, and linearly separable classification, as shown in Fig. 3. The size of each binary classification dataset is 1000 instances, divided into training, validation, and testing sets at a 6:2:2 ratio. The comparison is between our designed ANA and the identity function (no activation). As illustrated in Fig. 3b–e, various activation functions have distinct impacts on the decision boundaries in binary classification tasks, resulting in different levels of final model training accuracy. Sigmoid-type NAF (Fig. 3c) has the best classification accuracy (96%) on concentric circle datasets and the best classification accuracy (94.5%) on crescent moon datasets. ReLU-type NAF (Fig. 3b) has the best classification accuracy (89%) on linearly separable datasets. Figure 3f displays the learning curves for the three datasets. The results align with the widely accepted understanding that sigmoid-type activation functions perform well in binary classification tasks. This is primarily because the sigmoid-type function maps any real number to a range between 0 and 1, making their output highly suitable for interpretation as probabilities. However, owing to the shallow depth of our model, the nonlinear transformations introduced by the activation functions have a more direct and visible impact on the final decision boundary shape, resulting in its sharp angular features in the GSNR AF2’s decision boundary. Compared with GSNR AF2, GSNRs AF1 and 3 display smoother decision boundaries, leading to their gradual activation curve characteristics. Overall, GSNR AF2 is the best option for our network, achieving an average classification accuracy of 92.7% while maintaining high energy efficiency with a low threshold of 60 fJ.

**Fig. 4:**
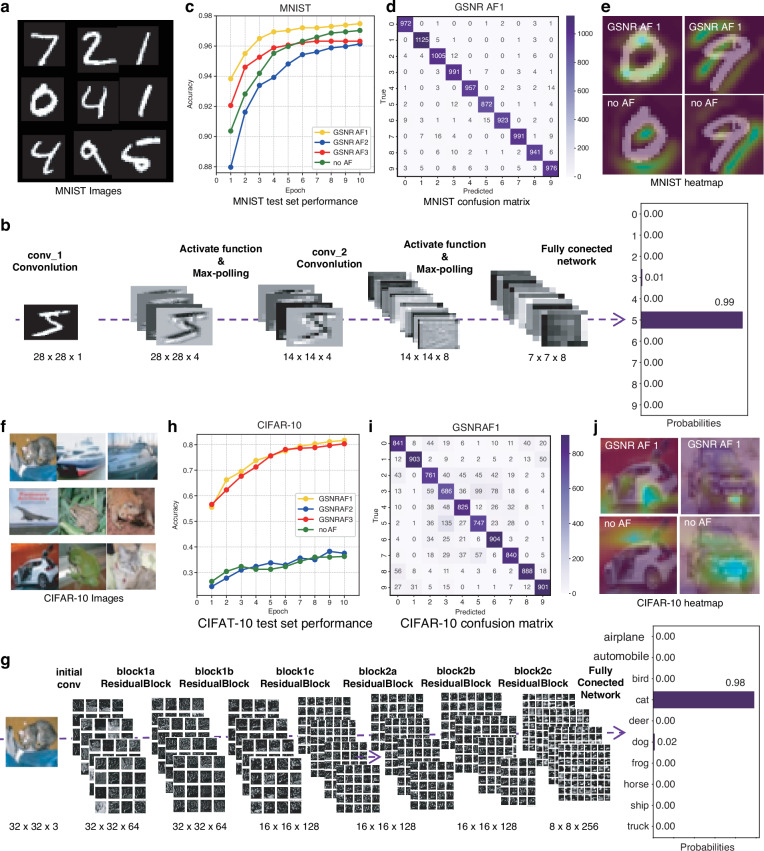
Performance comparison of NAFs on the MNIST and CIFAR-10 datasets. **a** Data examples of the MNIST test set. **b** Visualized activation map trained on the MNIST dataset with GSNR AF1. **c** Validation classification accuracy results using different activation functions on the MINST dataset. **d** Confusion matrix using GSNR AF1 on the MNIST dataset. **e** Heatmap comparison on the MNIST dataset between using GSNR AF1 and not using any activation function. **f** Data examples of the CIFAR-10 dataset. **g** Visualized activation map trained on the CIFAR-10 dataset with GSNR AF1. **h** Validation classification accuracy results using different activation functions on the CIFAR-10 dataset. **i** Confusion matrix using GSNR AF1 on the CIFAR-10 dataset. **j** Heatmap comparison on the CIFAR-10 dataset between using GSNR AF1 and not using any activation function

The on-chip picosecond pulse ONN not only works effectively on simple tasks such as binary classification tasks but also performs well in more complex image classification tasks. To solve these more challenging tasks, the spatiotemporal misalignment multiplexed picosecond pulsed optical neural network proposed above was used, as depicted in Fig. 3a. This architecture could significantly enhance device reusability and efficiency. Two neural networks are constructed via PyTorch for image classification tasks on the MNIST and CIFAR-10 datasets. The network structures are based on convolutional neural networks^64^ and residual networks^65^, and the details of the networks are illustrated in Figure S8 (see Section Ⅷ in the Supplementary Information). The raw input data samples are shown in Fig. 4a, f. Both datasets consist of ten classes and follow a standard class-balanced split: 40,000 images for training, 10,000 for validation, and 10,000 for testing. Comprehensive visualizations of the trained networks’ internal representations are provided in Fig. 4b, g. These figures offer an in-depth look at the output of each neural network block, with color coding representing activation intensities. This detailed representation allows for a holistic understanding of how information propagates through the network, from input to output, highlighting the transformations at each stage of the model.

To monitor the training process, the current model is evaluated on the validation set at each epoch, generating learning curves, as shown in Fig. 4c, h. When different activation functions are used to train a dataset, variations in accuracy occur due to their influence on the model’s nonlinear capabilities and gradient propagation. In both datasets, ReLU-type GSNR AF shows the best performance, with 97.53% classification accuracy in the MNIST dataset and 82.96% classification accuracy in the CIFAR-10 dataset. The confusion matrices for the test dataset images are presented in Fig. 4d, i, providing a comprehensive visualization of the models’ classification performance and highlighting potential areas of misclassification. Compared with the identity function (We compare its training results with those of several commonly used activation functions, and the results are presented in the Supplementary Information Section Ⅸ), GSNR AF1 demonstrated a 0.38% accuracy improvement on the MNIST test set and a 46.51% accuracy improvement on the CIFAR-10 test set. This substantial difference in accuracy improvement between the two datasets can be attributed to their inherent characteristics and complexity levels. The MNIST dataset consists of simple black-and-white handwritten digit images with relatively linear features. Consequently, a simple linear model could also achieve good classification results. In contrast, the CIFAR-10 dataset contains complex color images of objects that exhibit greater intraclass variations and a more intricate feature space, which requires more robust nonlinear feature extraction capabilities. Accordingly, ReLU-type GSNR AF demonstrates a significant advantage on the CIFAR-10 dataset because it effectively captures and represents complex nonlinear relationships in the data, such as the interactions between object shapes, textures, and colors. The heatmaps in Fig. 4e, j illustrate the networks’ activation patterns across different image regions. Models employing ReLU-type activation functions effectively highlight key features extracted by convolutional layers, such as areas potentially corresponding to car wheels, license plates, and the circular contours of digit ‘0’. In contrast, although a model without an NAF can detect simple features such as the central void in digit ‘0’, it struggles to effectively learn and emphasize more complex features of the car. In conclusion, GSNR AF1 demonstrates remarkable versatility by effectively capturing nonlinear features, thereby significantly enhancing the model’s classification accuracy and feature extraction capabilities across diverse datasets.

From the above model results, different tasks require distinct optimal NAFs, which emphasizes the need for reconfigurable ANAs (see Section Ⅸ in the Supplementary Information). Furthermore, to evaluate the practical performance of our picosecond optical pulse neural network architecture, we theoretically projected its classification capability on the MNIST dataset in Section Ⅹ in the Supplementary Information. Using optimistic yet reasonable estimation methods, our architecture demonstrates the potential to achieve a computational density of 2.13 × 10³ TOPS/mm² and an energy efficiency density of 0.71 × 10⁶ TOPS/W/mm² within a compact 4.15 mm² chip area, revealing the promising potential of all-optical neural networks compared to conventional electronic approaches.

## Discussion

In this work, we demonstrated femtojoule threshold reconfigurable graphene-silicon PhC cavity ANAs and proposed an on-chip wavelength division picosecond pulsed optical neural network for accurate and energy-efficient classification tasks. By inducing cavity-enhanced Kerr nonlinearity in silicon, multiple types of NAFs have been constructed in a silicon PhC cavity for the first time. The reconfigurable ANAs could obtain different types of nonlinear transmission responses at different specific wavelengths within a resonant peak. Additionally, by leveraging the slow light effect of the PhC, the optical pump efficiency can be increased, allowing for a reduction in the size of the ANA to 15 μm and an energy threshold of 300 fJ. To achieve a lower power threshold and faster response speed, we effectively combined the saturable absorption properties of graphene with the silicon PhC cavity, realizing an ultralow threshold energy below 100 fJ and a sub-5-ps ultrafast response, with the device’s optimal performance reaching a record 4-fJ power threshold and 1.05-ps response time. To expand on this concept, a deep learning neural network tailored for ANA is constructed, incorporating different forms of NAFs into a neural network computing model, and successfully applied to binary and image (MNIST and CIFAR-10) classification tasks via sigmoid-type and ReLU-type functions. Compared with networks without NAFs, this network achieves significantly lower power consumption and higher accuracy.

In conclusion, the graphene-silicon photonic crystal cavity all-optical nonlinear activator developed in this study simultaneously achieved femtosecond-level ultra-low operating threshold, picosecond-level ultrafast response speed, and dynamically reconfigurable characteristics, providing a key functional unit for building high-performance integrated optical computing systems. This technological breakthrough demonstrates the great potential of photonic computing in achieving energy-efficient neural network computing, laying an important foundation for developing new computing paradigms for artificial intelligence applications.

## Materials and methods

### Device fabrication and measurement

The fabrication flowchart and measurement are described in detail in Section Ⅳ in the supplementary information.

## Supplementary information

Supplementary Material for Femto-joule threshold reconfigurable all-optical nonlinear activators for picosecond pulsed optical neural network

## Data Availability

All the data supporting this study are available in the paper and Supplementary Information. Additional data related to this paper are available from the corresponding authors upon request.
