# Supplementary material for: Femto-joule threshold reconfigurable all-optical nonlinear activators for picosecond pulsed optical neural networks
Source: Light Sci Appl. 2026 Feb 27;15:128. doi: 10.1038/s41377-025-02175-4 (PMC12946305; doi:10.1038/s41377-025-02175-4)
Supplement: Supplementary file 1 — Supplementary Material for Femto-joule threshold reconfigurable all-optical nonlinear activators for picosecond pulsed optical neural network [file 41377_2025_2175_MOESM1_ESM.docx]

**Supplementary Information**

**Femto-joule threshold reconfigurable all-optical nonlinear activators for picosecond pulsed optical neural networks**

**Ruizhe Liu^1,†^, Zijia Wang^1,†^, Chuyu Zhong^1,2,†^, Yan Chen^1,3,†^, Boshu Sun^3,4^, Jialing Jian^3,4^, Hui Ma^1^, Dawei Gao^5^, Jianyi Yang^1^, Lan Li^3,4, 6,*^, Kaihui Liu^7,*^, Xiaoyong Hu^7,*^, Hongtao Lin^1,5,*^**

*^1^ The State Key Lab of Brain-Machine Intelligence, Key Laboratory of Micro-Nano Electronics and Smart System of Zhejiang Province, College of Information Science and Electronic Engineering, Zhejiang University, Hangzhou 310027, China*

*^2^Shenzhen Technology University, College of Integrated Circuits and Optoelectronic Chips, Shenzhen 518118, China*

*^3^Zhejiang Key Laboratory of 3D Micro/Nano Fabrication and Characterization, Westlake Institute for Optoelectronics, Fuyang, Hangzhou, Zhejiang 311421, China*

*^4^ Zhejiang Key Laboratory of 3D Micro/Nano Fabrication and Characterization, School of Engineering, Westlake University, Hangzhou, Zhejiang 310030, China*

*^5^College of Integrated Circuits, Zhejiang University, Hangzhou 310027, China*

*^6^Institute of Advanced Technology, Westlake Institute for Advanced Study, Hangzhou, Zhejiang 310024, China*

*^7^State Key Laboratory for Mesoscopic Physics, Frontiers Science Center for Nano-optoelectronics, School of Physics, Peking University, Beijing 100871, China*

This Supplementary Information comprises the following sections:

Section Ⅰ - Measurement setups

Section Ⅱ - Design of the transmittance of photonic crystal cavity devices

Section Ⅲ- Optical resonant cavity model of a silicon all-optical nonlinear activation function device

Section Ⅳ - Fabrication process flow, morphology of our devices and material properties of the graphene

Section Ⅴ - Details of the performance of the graphene-silicon device

Section Ⅵ - Details of the picosecond pulse optical fully connected neural network architecture

Section Ⅶ - Details of the optical activation functions

Section Ⅷ - Details of the optical neural network and datasets

Section Ⅸ - Results comparison of the networks using different activation functions

Section X - Prospective Performance Evaluation of the On-Chip Picosecond-Pulsed Optical Neural Network (Based on the performance of ANA)

**Section Ⅰ - Measurement setups**

The characterization setup is shown in detail in Fig. S1. The femtosecond pulse source at a central wavelength of 1550 nm is shipped with the FLS1950F-CUSTOM laser (pulse width: ∼100 fs, repetition rate: 50 MHz) and amplified with EDFA100P. The continuous wavelength source at a central wavelength of 1550 nm was an Agilent 8164A measurement system. Fig. S1a shows the transmission spectrum and saturable absorption measurement system, where the power of incident and outgoing waves is measured by means of two optical power meters (PMs), and then the transmission of the device $T=\frac{P_{out}}{P_{in}}$ is determined, resulting in the experimental nonlinear transmission curve as a function of input power, as shown in Fig. S1c shows. More specifically, we first obtain the grating coupling efficiency $\eta$ from the spectrum of the reference device by 10log ($\eta^{2}$)=dB, and DAQ provides a voltage to control the change in VOA, modulating $P_{in}$, where $P_{in}=PM1*\eta$. Additionally, $P_{out}=\frac{PM2}{\eta}$.


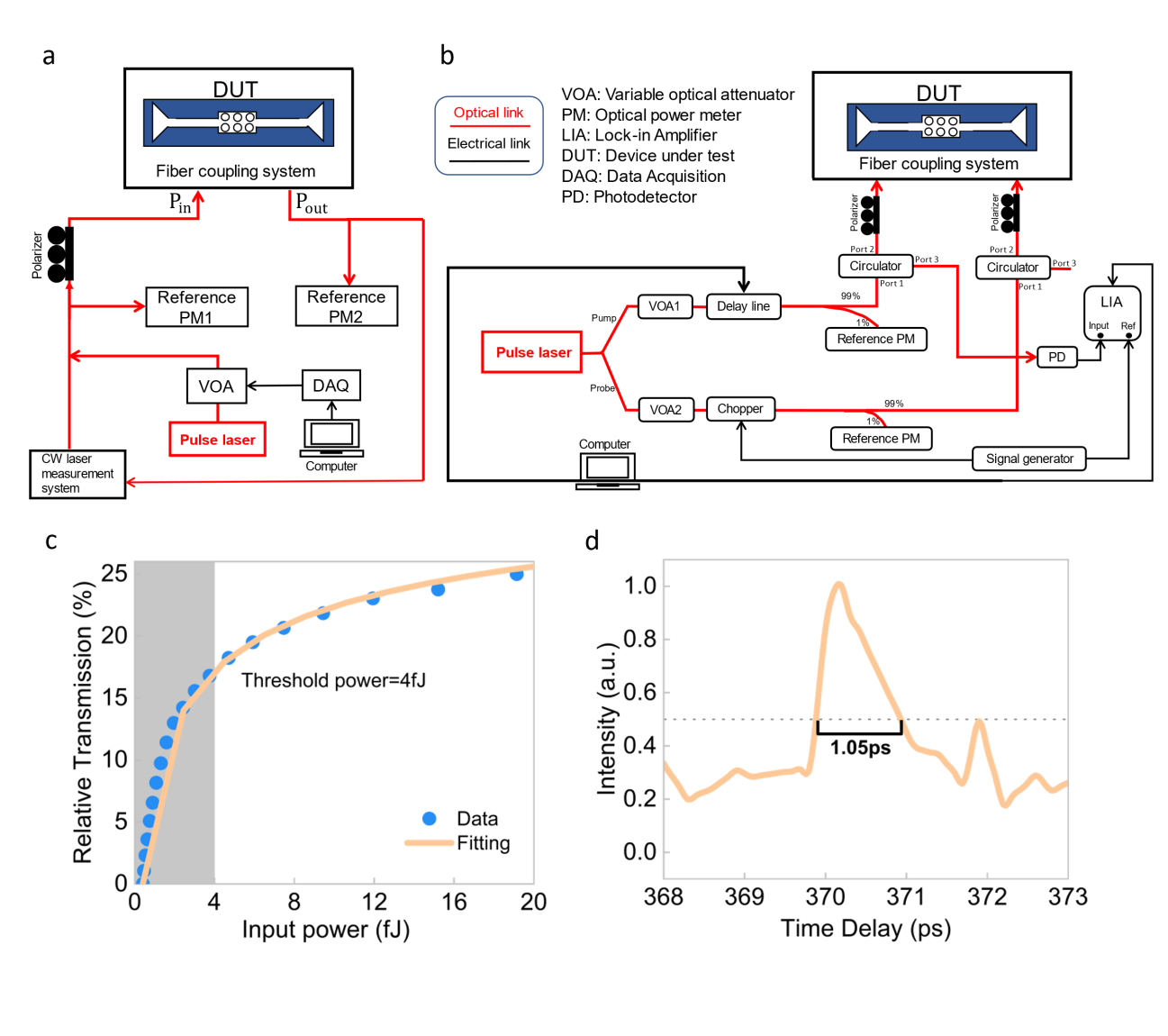


**Fig. S1 | Schematic of our measurement** **setup.** **a.** Schematic of the saturable absorption measurement system. **b.** Schematic of the pump-probe measurement system. The 1550 nm femtosecond laser is divided into two paths (pump light and probe light). VOA: Variable optical attenuator, PM: Optical power meter, DUT: device under test. **c.** Example of saturable absorption measurement. **d.** Example of a pump-probe measurement.

We also explore devices with the pump-probe measurement setup shown in Fig. S1b. The femtosecond laser is divided into two paths (pump light and probe light). The pump light is modulated by a time delay line, which supplies a strong signal for exciting electrons and causes the photobleaching effect in graphene, whereas the probe light is weak, which is modulated by a chopper to be easily detected by a lock-in amplifier. Two signals illuminate the same area of the device with a time delay. If the time delay exceeds the relaxation time of the device, the excited electrons return to the valence band from the conduction band, and the probe light is subsequently absorbed by the graphene and changes the band structure. However, if it is within the relaxation time, the probe light will cross the band directly without exciting electrons, leading to enhanced transmission. The response time is obtained by fitting the peak read from the lock-in amplifier, as shown in Fig. S1d. To gain a deeper understanding of these properties at different wavelengths, a filter is added after the laser, converting femtosecond light sources into picosecond light sources with specific input bandwidth wavelengths.

**Section Ⅱ -** **Design of the transmittance of photonic crystal cavity devices**

The parameter design and simulation of the transmittance of photonic crystal cavity devices are demonstrated in this section. We demonstrate two different kinds of photonic crystal devices, one of which is a silicon resonant cavity device^1,^ and the other is a graphene-silicon hybrid device. The photonic crystal of this silicon - based device has a period of 0.35 μm, a duty cycle ranging from 0.25 to 0.35, and is arranged in a triangular lattice. The width of the line defect is 0.38 μm. The cavity in our device is formed into a one - dimensional resonant cavity structure, with the duty cycle gradually decreasing from the edge to the center of the device. As a result, light pulses resonate between the mirrors at the edge of the photonic crystal. The scanning electron microscope (SEM) image of the device (Fig. S2a) clearly shows the size difference between the edge holes and the central holes, providing evidence for this unique cavity structure. Fig. S2b shows the normalized transmission spectrum of the photonic crystal waveguide. There are four resonance peaks in this transmission spectrum.


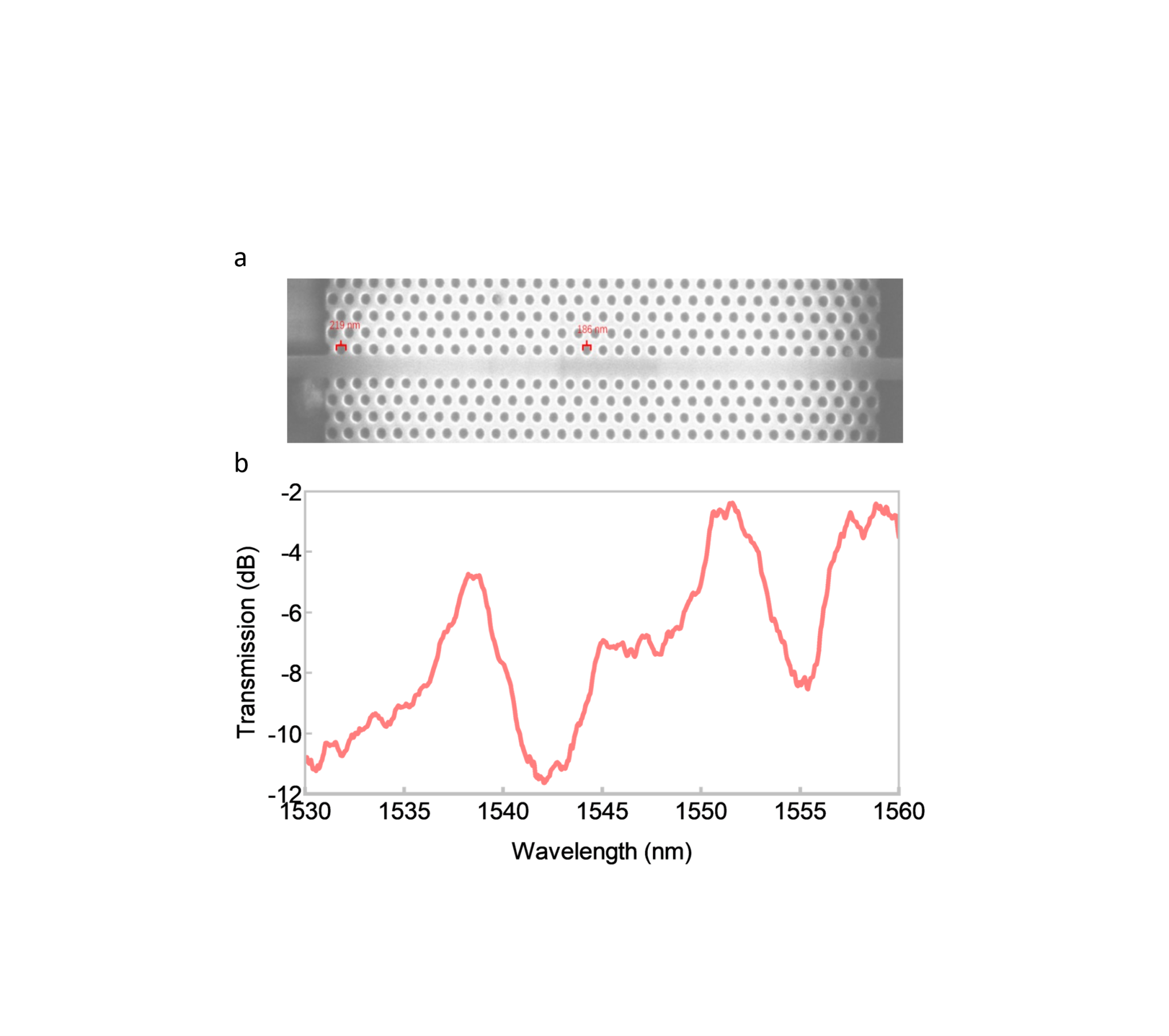


**Fig. S2 |** **a.** The SEM image of the top view of the silicon PhC cavity ANA, whose edge holes are larger than those in the central part. At the edge, mirrors are formed to reflect the input light and thus form a resonant cavity structure. **b.** Normalized transmission spectrum of PhC cavities: We designed specific resonant peaks to enhance the interaction between the pump light and our silicon photonic crystal cavities.

**Section Ⅲ - Optical resonant cavity model of a silicon all-optical nonlinear activation function device**

Here, we develop a simple analytical model of an optical resonant cavity to describe our nonlinear activation device. The repetition rate of our laser is labeled $f$, the pulse width is $\tau$, and the energy of a single pulse is $E_{pulse}$. When our average input power is denoted as $P_{average}$, the peak input power is therefore:

$$\begin{aligned} P_{peak} = \frac{E_{pulse}}{\tau}=\frac{P_{average}}{f\tau}\#\left( S.1 \right) \end{aligned}$$

Moreover, the shift in the probe resonance wavelength of our photonic crystal cavity follows classical cavity perturbation theory^2^:

$$\begin{aligned} \Delta\lambda_{kerr}=\lambda_{0}\cdot\frac{\int_{c} n_{2}\left( \boldsymbol{r} \right){\left| E_{0}\left( \boldsymbol{r} \right) \right|^{2}n\left( \boldsymbol{r} \right)\varepsilon}_{0}\left| E_{0}\left( \boldsymbol{r} \right) \right|^{2}dV}{\int_{c} \varepsilon_{c}\left| E_{0}\left( \boldsymbol{r} \right) \right|^{2}dV}\#\left( S.2 \right) \end{aligned}$$

where $\boldsymbol{r}$ represents the position vector, $\lambda_{0}$ represents the probe resonant wavelength, $n$ corresponds to the refractive index, and $n_{2}$ represents the third-order nonlinear coefficient of the cavity material. $\varepsilon_{0}$ is the vacuum permittivity, and $\varepsilon_{c}$ is the spatially dependent cavity dielectric constant distribution. $E_{0}$ is the electric field complex amplitude of the cavity mode.

Additionally, when we assume that the photonic crystal resonant cavity has a quality factor $Q$, the total energy stored in the cavity that inputs the cavity is given by^3^:

$$\begin{aligned} W=\frac{\varepsilon_{0}}{2}\int_{c} \varepsilon_{c}\left| E_{0} \right|^{2}dV=\frac{QP_{peak}}{2\omega}\int_{c} \varepsilon_{c}\left| E_{N} \right|^{2}dV\#\left( S.3 \right) \end{aligned}$$

where $P_{peak}$ is the pump pulse peak power coupled into the cavity, $\omega$ is the angular frequency of the pump light, and $E_{N}$ represents the dimensionless eigenmode of the photonic crystal cavity, which is normalized so that $\int_{c} \left| E_{N} \right|^{2}dV=1$, where the integration is carried out through the whole cavity. When the pump light is coupled into the cavity, the total energy can be calculated as the power being magnified $Q$ times.

The next part is concerned with the Kerr optic resonance detuning of our cavity. According to cavity perturbation theory, considering the optic resonance detuning corresponding to the refractive index shift caused by the Kerr effect, a simplified expression can be correlated with the waveguide effective index change and group index, which can be calculated as^2,4^:

$$\begin{aligned} \Delta\lambda=\frac{\Delta n_{eff}}{n_{g}}\cdot\lambda_{0}.\#\left( S.4 \right) \end{aligned}$$

where $\Delta n_{eff}$ denotes the waveguide effective index change due to the change in the material index caused by the Kerr effect and where $n_{g}$ denotes the model group index. According to the definition of the third-order nonlinear refractive index^5^:

$$\Delta n=n_{2}\left| E_{0} \right|^{2}$$

We can determine the effective index change of our cavity, which is influenced by the electric field in the cavity, as follows:

$$\begin{aligned} \Delta n_{eff}=\sum_{j} {n_{2}}_{j}{\left| E_{0} \right|^{2}}_{j}\Gamma_{j}={n_{2}}_{eff}\cdot QP_{peak}\cdot n_{g}\#\left( S.6 \right) \end{aligned}$$

where $j$ denotes different parts of the waveguide, $\Gamma$ represents the confinement factor of the waveguide^6^, and ${n_{2}}_{eff}$ is the effective third-order nonlinear coefficient of the waveguide calculated by combining the weighted average of the third-order nonlinear coefficients of the waveguide material and Eq. S.3:

$$\begin{aligned} {n_{2}}_{eff}\cdot QP_{peak}=\sum_{j} {n_{2}}_{j}\cdot\frac{\gamma_{j}}{n_{j}}\cdot{\left| E_{0} \right|^{2}}_{j}\#\left( S.7 \right) \end{aligned}$$

where $\gamma$ represents the spatial confinement of energy density^6^.

Finally, we can derive a simplified equation that depicts the resonance detuning of the cavity:

$$\Delta\lambda=\frac{\Delta n_{eff}}{n_{g}}\cdot\lambda_{0}={n_{2}}_{eff}\cdot QP_{peak}\cdot\lambda_{0}$$

**Section Ⅳ - Fabrication process flow, morphology of our devices and material properties of the graphene**

Device fabrication was based on the SOI platform. Our devices were fabricated on an SOI wafer with a 220 nm thick device layer and a 2 µm thick SiO2 box layer. The main steps are illustrated in Fig. S3. First, the photonic crystal line defect waveguide structures were patterned via electron beam lithography (EBL) and etched via an inductive coupling plasma (ICP) process to a full depth of 220 nm (steps ⅰ-ⅳ). Second, the CVD graphene on the copper substrate was transferred onto the devices via the wet-transfer method^7^ (step ⅴ). The loaded graphene was then patterned via EBL, and the unwanted part was etched via oxygen plasma (steps ⅵ-ⅷ). Finally, a graphene‒silicon hybrid device was fabricated.


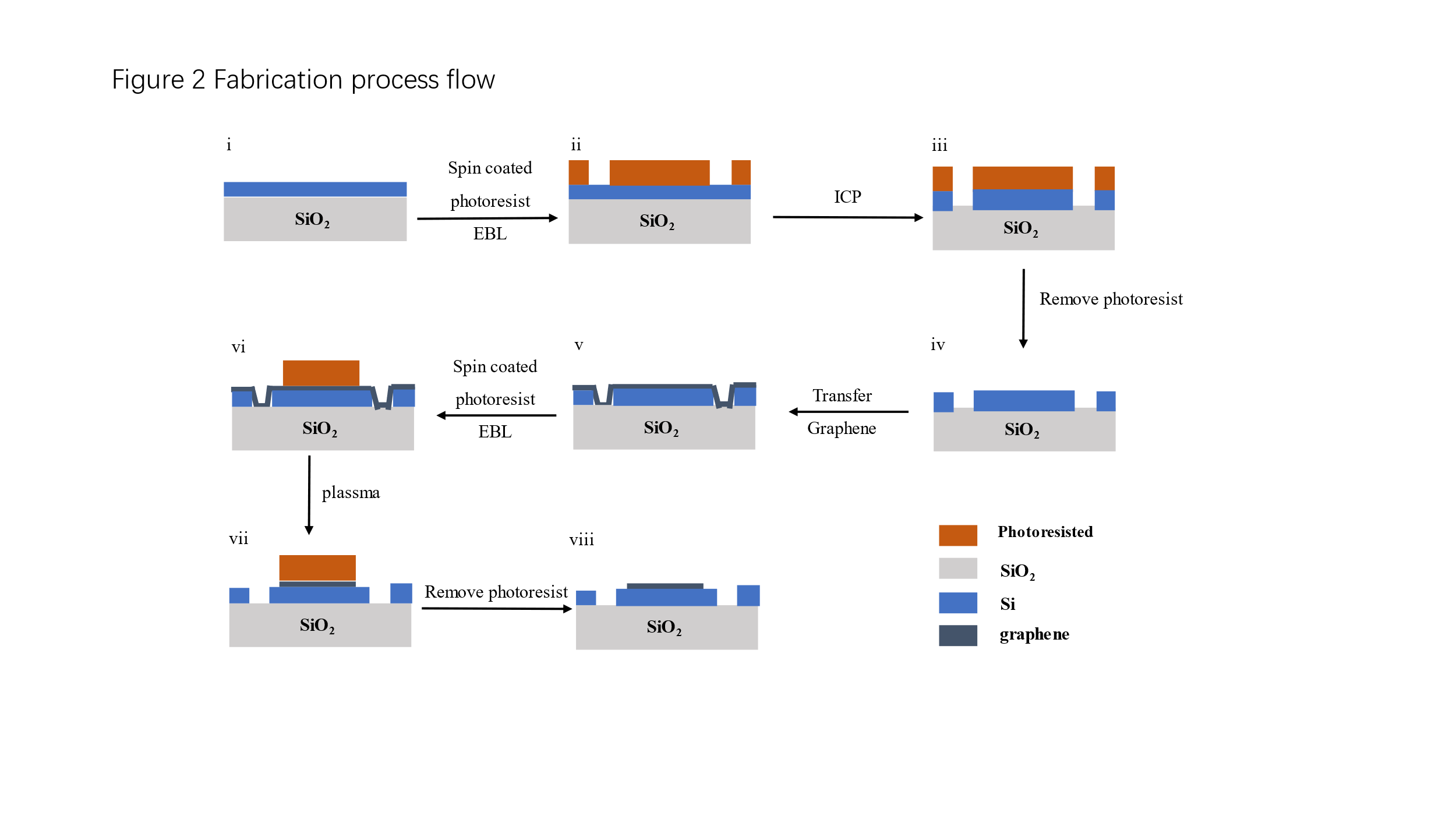


**Fig. S3 | Fabrication process flow.** **i:** original sample, **ii-iv:** waveguide fabrication, **v:** graphene transfer, **vi-vii:** graphene pattern, viii: final device after graphene patterning.

The material properties of the graphene samples used in our devices were characterized and demonstrated in this section. We performed Hall effect measurements (Ecopia HMS-5000) and Raman spectroscopy (Witec alpha300R). Graphene transferred to 300 nm thick silicon dioxide substrates and graphene on devices with photoresist cladding were characterized.

We used the van der Pauw method to perform Hall effect measurements under a magnetic field intensity of 0.535 T. The electric properties of the graphene on the silicon dioxide substrate are listed in Table S1. Low resistivity and high mobility were obtained. The sign of the sheet concentration indicates p-type doping of our sample.

**Table S1. Electrical properties of the graphene.**

| Sheet Concentration  (cm^-3^) | Sheet Resistance  (Ω/□) | Resistivity  (Ω·cm) | Conductivity  (S·cm) | Mobility  (cm^2^·Vs^-1^) |
| --- | --- | --- | --- | --- |
| 4.933×10^12^ | 1.1459×10^3^ | 1.1459×10^-4^ | 8.7271×10^3^ | 1.1043×10^3^ |


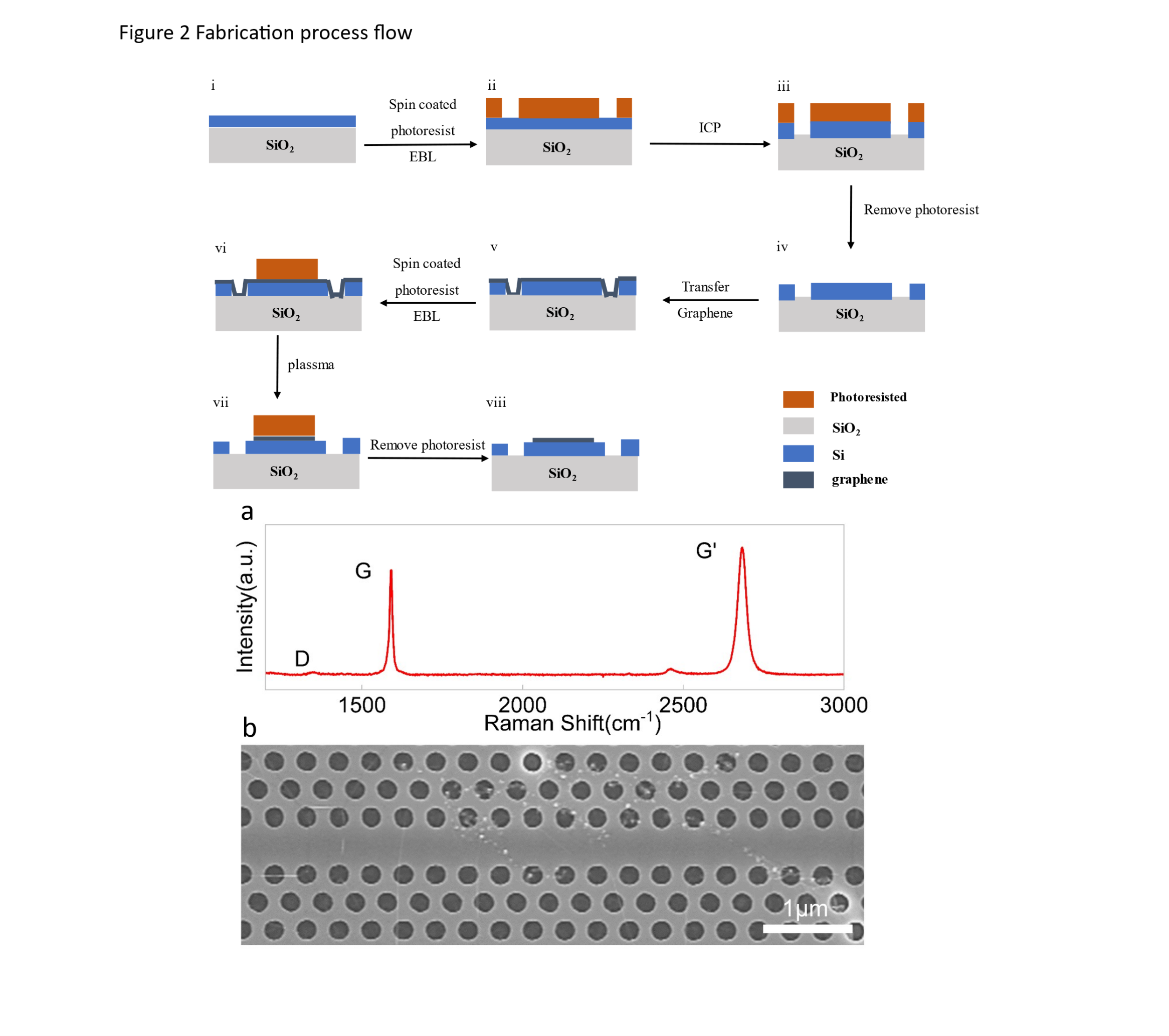


**Fig. S4 | Material properties of the graphene and morphology of the devices.** a. Raman spectra of graphene samples on the silicon oxide substrate, where different peaks are indicated. **b.** SEM image of the device with graphene.

As Fig. S4a shows that the intensities of the D peaks are very low, indicating that our graphene samples have few defects. In addition, the shape of the 2D peak is the most effective way to identify a single layer, indicating that our sample has only one layer. Here, we illustrate the morphology of our device shown in Fig. S4b. Clearly, graphene covers the photonic crystal and line defects on the device surface.

**Section Ⅴ - Details of the performance of the graphene-silicon** **device**

We measured the optical response of the graphene PhC device at different wavelengths. Taking advantage of silicon Kerr third-order nonlinearity effects, as discussed above, the nonlinear response of the graphene PhC device can be reconfigured.

When a wavelength near 1520 nm is selected on the basis of the spectrum, the device exhibits a downward-type activation function. The transmittance at 1522.5 nm gradually decreases with the redshift of the transmission spectrum, forming a downward-type activation function. When a wavelength near 1534 nm is selected, the device is reconfigured into an upward-type activation function. This is because the transmittance at 1534 nm is relatively stable, with a gradual redshift in the transmission spectrum, leading to a minimal impact on the transmittance from the redshift of the transmission spectrum after pulse light is injected. This indicates that the saturable absorption of graphene remains the primary influencing factor, resulting in an upward-type activation function. When a wavelength near 1541 nm is selected, the device is reconfigured into a ReLU-type activation function. This is because the transmittance at 1541 nm first gradually decreases and then counteracts the increasing trend of saturable absorption. After reaching the minimum value, it gradually increases and superimposes with the upward trend of saturable absorption, forming a ReLU-type activation function. Pump-probe measurements of the response speed of various activation functions were also conducted, with full width at half maximum response times of 4.38 ps, 4.03 ps, and 4.32 ps for the three activation functions.


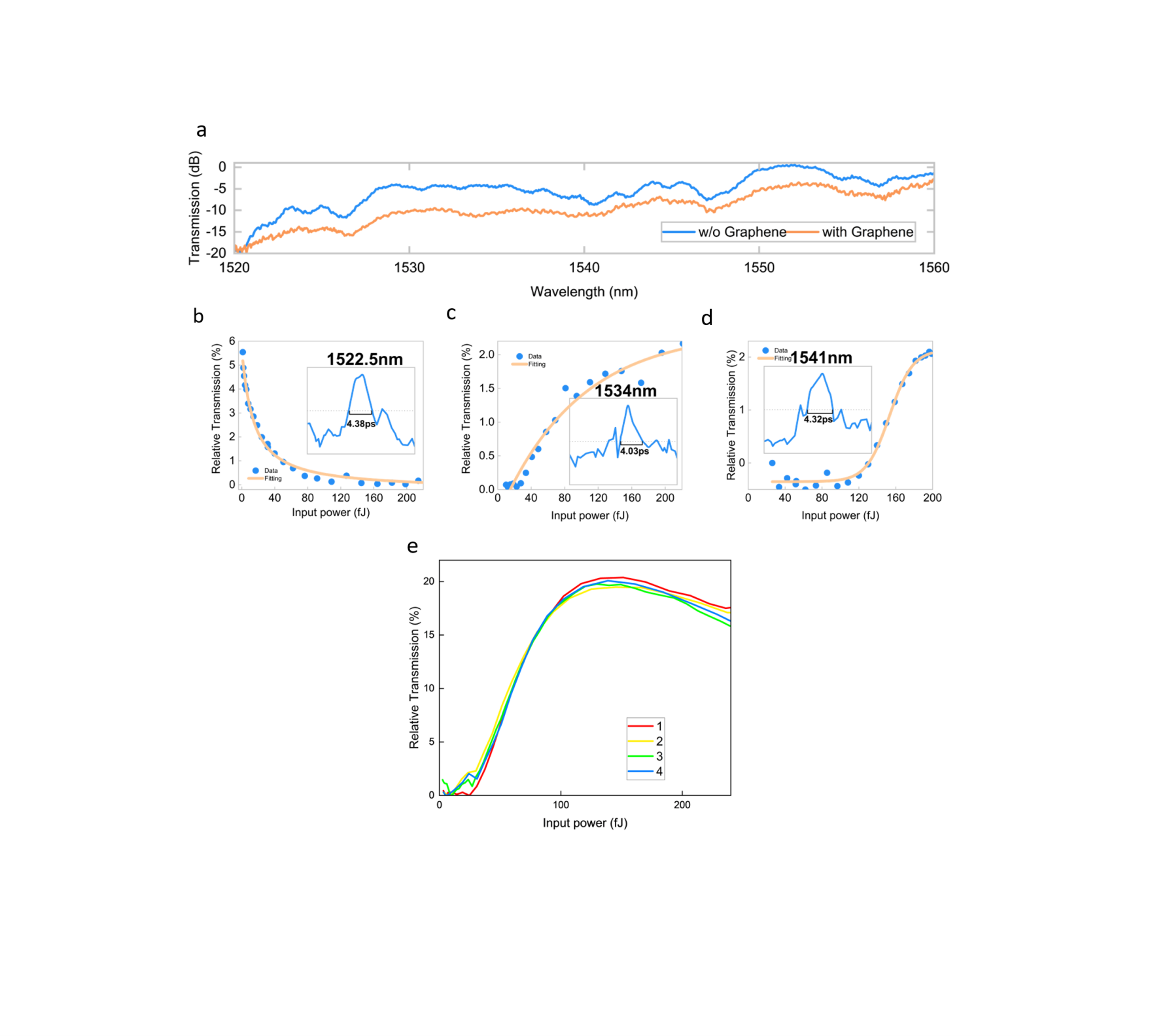


**Fig. S5 | Supplementary properties and performance of the graphene-silicon integrated NAF device. a.** Normalized transmission spectra of a PhC ANA without graphene (blue curve) and with graphene (orange curve). **b-d.** Reconfiguration activation functions at different wavelengths. The inset shows the time delay measurement for the response time at different wavelengths. **e.** Four tests of the nonlinear activation function curve of the graphene-silicon integrated ANA device under the pumping of the input light with a wavelength of 1540 nm.

To verify the remarkable stability and repeatability of the proposed graphene - silicon integrated ANA device, Figure S5e presents the nonlinear activation function curve of this device under the pumping of 1540 - nm input light. As depicted in the figure, four consecutive tests were carried out on the nonlinear activator. Evidently, the activation function generated by the activator demonstrates excellent repeatability and stability.

**Section Ⅵ -** **Details of the picosecond pulse optical fully connected neural network architecture**

The entire fully connected binary classification optical neural network consists of a 4-channel signal loading layer, three 4×4 fully connected layers with picosecond-response nonlinear activation capability, and an output layer, as shown in Fig. S6a. First, a picosecond light source is generated by a femtosecond laser in the four-channel signal loading layer and then coupled into the on-chip system. The pulses are next split into four beams with different wavelengths through an ID-WDM device and then encoded by four high-speed modulators. Then, the pulses are combined through an ID-WDM into a waveguide and sent to the next 4×4 fully connected layer. In the next layer, the pulses are distributed to four different neurons for processing. Each neuron is divided into synapses and activations. The pulses are sent to the ID-WDM and split into different wavelengths, weighted differently and finally combined into one channel. Next, the pulses pass through an ANA, and new pulses are nonlinearly activated to be combined with other neurons’ output pulses through an ID-WDM. The pulses are transmitted to the next fully connected layer of the network. After three fully connected layers, the pulses are sent to the output layer for the last weight operations and activations and then sent directly to a high-speed detector for signal output.

Although the architecture in Figure S6a activates at different wavelengths by using the wavelength division multiplexing technology, it doesn't mean that the activation functions at different wavelengths are completely different. The wavelengths used in our architecture can be chosen as consecutive multiple wavelengths within the same band. Since the variation trend of the transmission spectrum of the device doesn't change within the same band, the activation functions generated in this band are basically similar. In Figure S6. (b), we present the activation function curves at different wavelengths within the 1533 - 1537 nm band. It can be seen that these activation functions have a uniform variation trend, which supports the normal operation of our architecture.


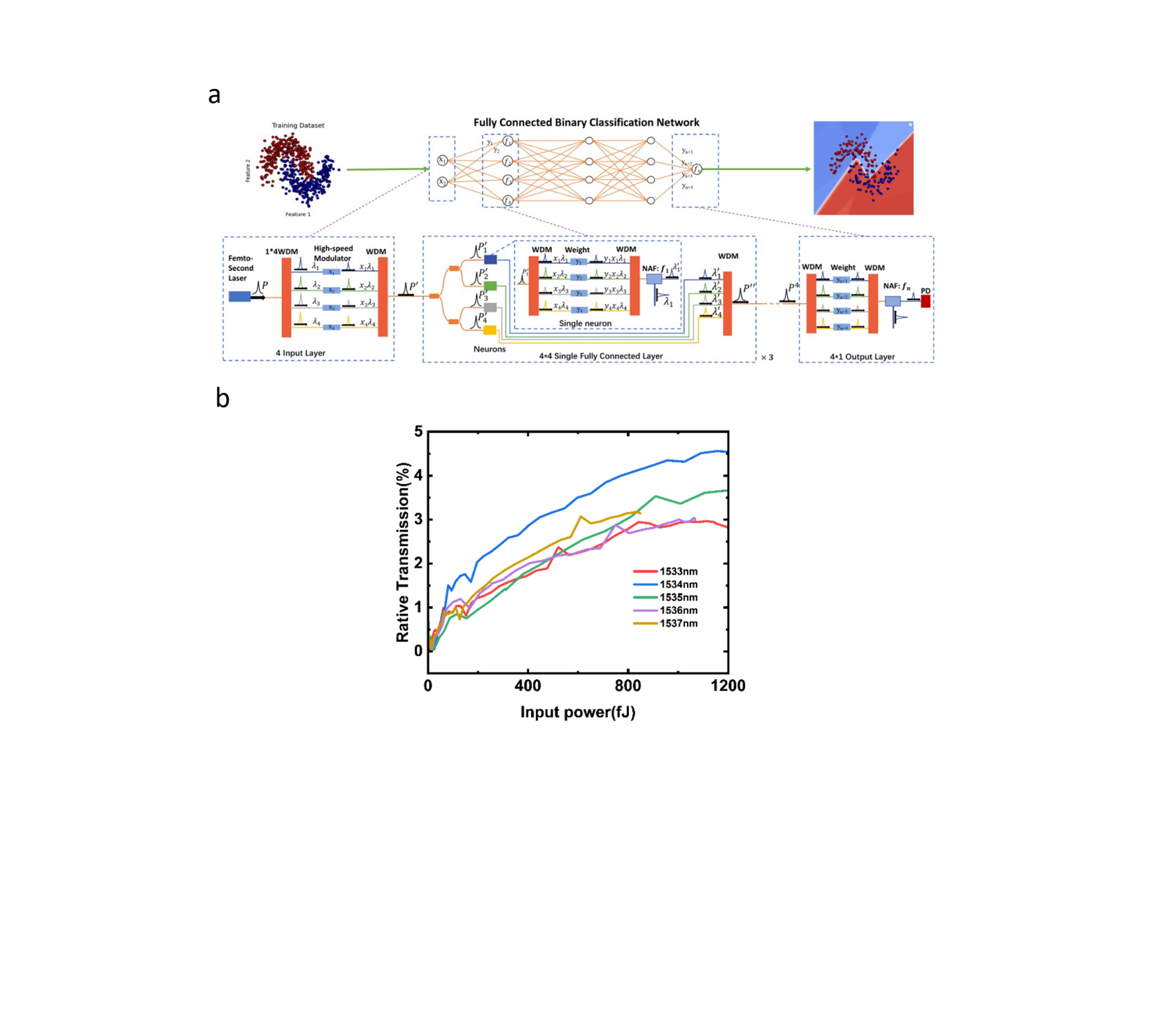


**Fig. S6 |** **a.** General block diagram of a fully connected binary classification optical neural network, which consists of a 4-channel signal loading layer, three 4×4 fully connected layers with picosecond-response nonlinear activation capability, and an output layer. **b.** Activation functions at different wavelengths within the 1533 - 1537 nm band. It can be seen that activation functions of similar wavelengths exhibit similarities.

Section Ⅶ - Details of the optical activation functions

According to the network structure and device that have already been constructed, it is necessary to generate a nonlinear activation function with the reconfigurable silicon-graphene ANA.

First, the nonlinear response generated by our ANA is fitted into an NAF curve through a linear fitting method. For the activation functions, as the data come in discrete points that are not continuous, an interpolation has to be made to allow for network training. However, the points are more than necessary to obtain a well-fit interpolated piecewise function, and a piecewise function with too many points slows neural network training by a significant portion; thus, some optimization is needed. As cubic spline interpolation was unable to adequately capture the trend of points, we adopted a linear interpolation method to fit the curve. This decision, while unconventional, was made with the understanding that linear interpolation would result in a fitted curve that is not differentiable everywhere. However, the experimental results (in Section X) demonstrated that this approach had minimal impact on the model's backpropagation process. Remarkably, the model incorporating the linearly interpolated activation function still exhibited excellent classification performance. Consequently, we retained this method for function fitting.

The fitted curves were subsequently processed, including normalization and zero-point adjustment. Normalizing and adjusting the zero point of the fitted curve are performed to make its characteristics more similar to those of classic activation functions, such as ReLU or sigmoid. This process helps improve the model's performance and stability. The normalization operation scales the output range of the curve to a standard interval to help prevent the explosion or vanishing of activation values, thereby increasing the stability of network training. Zero-point adjustment can help the network better handle positive and negative inputs and produce ReLU-type or sigmoid-type sparse activation characteristics near $x=0$. Through these processes, our custom activation function can maintain its unique nonlinear characteristics while also possessing some advantageous features of classic activation functions, thus performing better in neural networks. The expressions of the five optical activation functions are shown below.


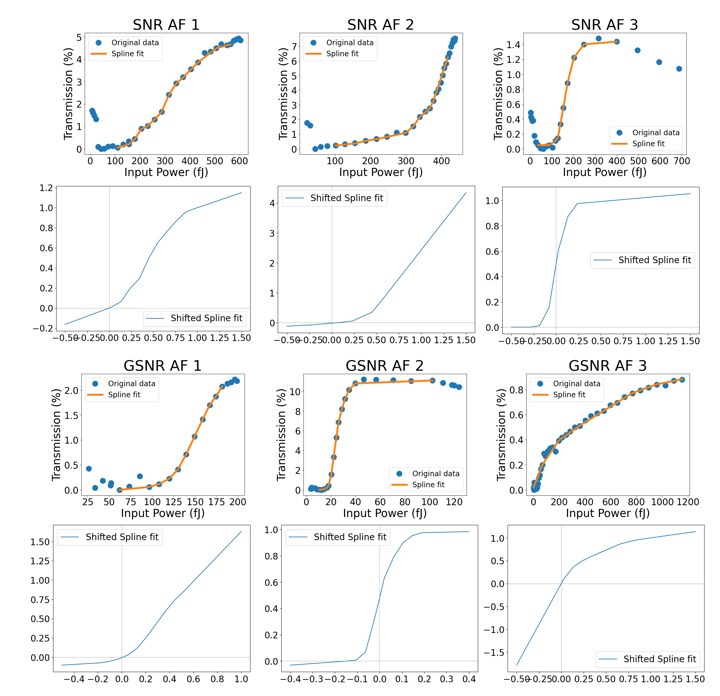


**Fig. S7 |** Original data and fitted and calibrated curves for reconfigurable silicon/silicon-graphene ANAs. For each subplot, the blue dots represent the original data points; the red dashed lines represent the fitted curves; and the green solid lines represent the calibrated curves after normalization and zero-point adjustment.

**Section Ⅷ -** **Details of the optical neural network and datasets**

In this work, a total of 5 datasets and 3 network architectures are employed, as depicted in Fig. S8, to investigate the efficacy of activation functions when facing different tasks and models. Among these, 3 binary classification datasets are generated utilizing the make-moons, make-circles, and make-classification functions from the sklearn library. Each of these datasets is classified by the fully connected network, and the architecture is depicted in Fig. S8a. The MNIST dataset is classified by the convolutional neural network (CNN), as illustrated in Fig. S8b. The CIFAR-10 dataset is classified by the residual network (ResNet), as illustrated in Fig. S8c. Furthermore, in acknowledging the transmission constraints inherent to optical neural networks, we have constrained the floating-point numbers to a precision of four decimal places.


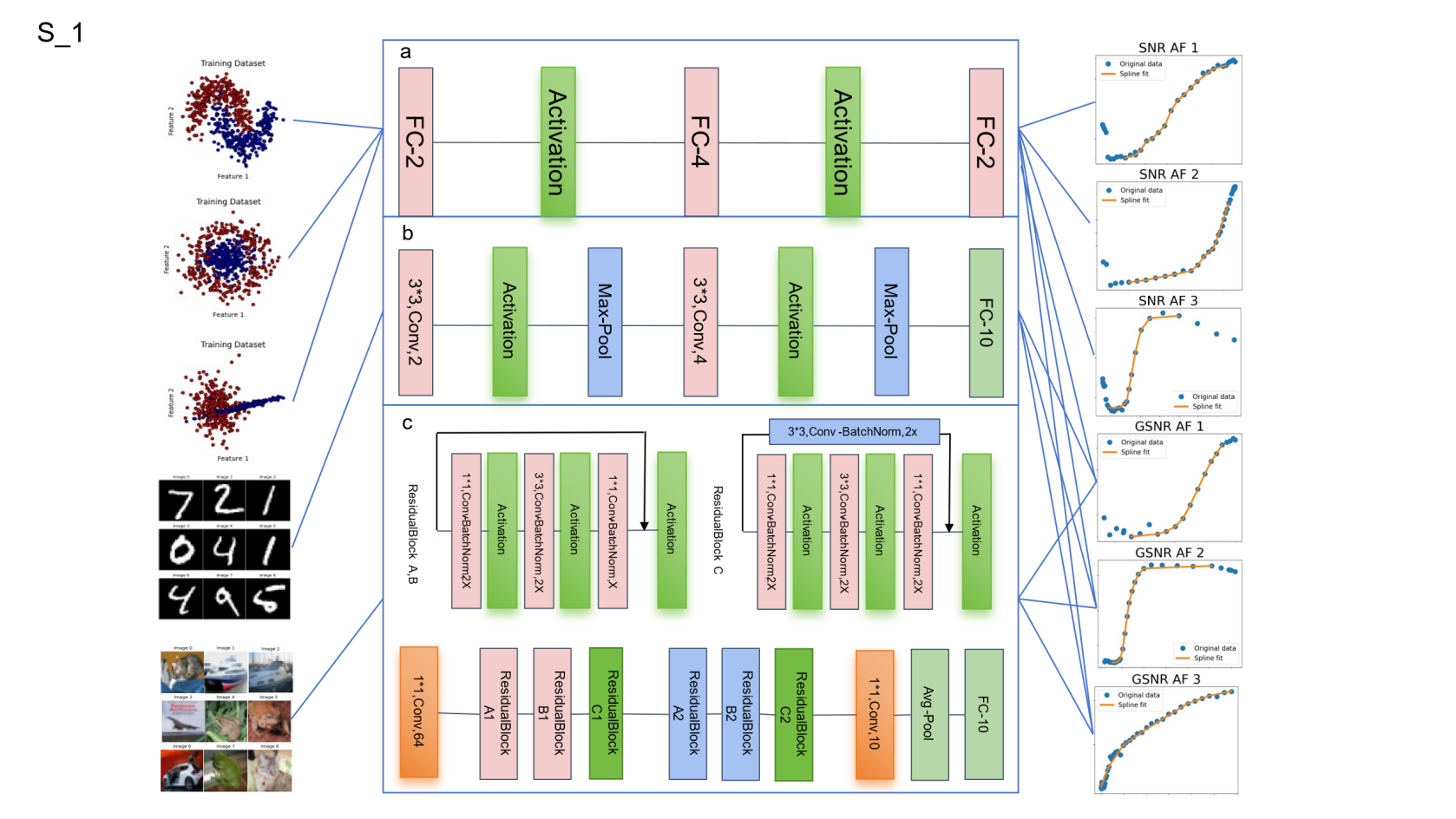


**Fig. S8 | Architectures of the models with their respective datasets and activation functions. a.** Binary classification datasets classified by the fully connected network via silicon nonlinear response activation functions (SNRs AFs). **b.** MNIST dataset classified by the CNN via graphene/silicon heterojunction nonlinear response activation functions (GSNR AFs). **c.** CIFAR-10 dataset classified by ResNet via graphene/silicon heterojunction nonlinear response activation functions.

To evaluate the performance of different activation functions, we replace the activation function in the model accordingly. Initially, fitting the nonlinear responses generated by optical devices on the basis of experimentally collected data is essential. While several fitting techniques are available, such as linear fitting, polynomial fitting, and cubic spline fitting, this study opts for linear fitting due to the large number of data points used, aiming to approximate the nonlinear curves accurately. Moreover, to enhance convergence and improve performance during the training process, normalization was applied to the sampling points, and the relative reference point was adjusted. In practical applications within optical networks, this adjustment can be achieved by modulating the phase and amplitude of the light.

For the fully connected network used for binary classification, the proportions of the training, testing, and validation sets are 6:2:2, respectively. Training commences with a 0.01 learning rate, the Adam optimizer, the BCELoss function and the network trained on the entire dataset for 100 epochs. For the CNN utilized for the MNIST dataset, the training of the model started at a 0.001 learning rate, Adam optimizer, cross-entropy loss function and 100 batch size. The CNN trained for 10 epochs. For ResNet applied to the CIFAR-10 dataset, the training of the model started with a 0.001 learning rate, the Adam optimizer, the cross-entropy loss function and a 128 batch size. ResNet was trained for 10 epochs. Both the MNIST and CIFAR-10 datasets comprise ten classes with a standard class-balanced 40,000 training, 10,000 validation, and 10,000 test images. All the above models are trained on a single NVIDIA RTX 4090 GPU.

**Section Ⅸ -** **Results comparison of the networks using different activation functions**

In this work, we involved five datasets and three networks, providing detailed experimental results. The extensive use of various datasets and network architectures to evaluate the performance of activation functions aims, on the one hand, to emphasize the wide applicability of activation functions generated by optical devices and, on the other hand, to highlight the importance of the reconfigurability of the devices. This implies that in practical optical neural networks, our devices are not only capable of accommodating multiple classification tasks but can also generate a variety of responses through the special design of the transmission spectra of silicon devices and the transfer of graphene materials, enhancing the practicality of the design.

Initially, we explored the impact of different activation functions on the classification results of binary datasets. To compare the effects of the activation functions, we included a network without any activation function and visualized all the results. As illustrated in Fig. S9, the classification effectiveness of the models integrated with the activation functions surpasses that of the model without any activation functions. This advantage seems to be due to the model's capacity to map learned complex target functions into high dimensions via nonlinear activation functions. Without the activation functions, the model can only linearly divide the datasets or even fail to converge (as shown on the far right side of Fig. S9). Different activation functions exhibit varying activation effects, thereby affecting the final model performance differently. For binary classification tasks, activation functions with a sigmoid-like shape exhibit better classification effectiveness; for more complex classification tasks, ReLU-type activation functions offer stronger capabilities to counteract gradient vanishing in deep models.


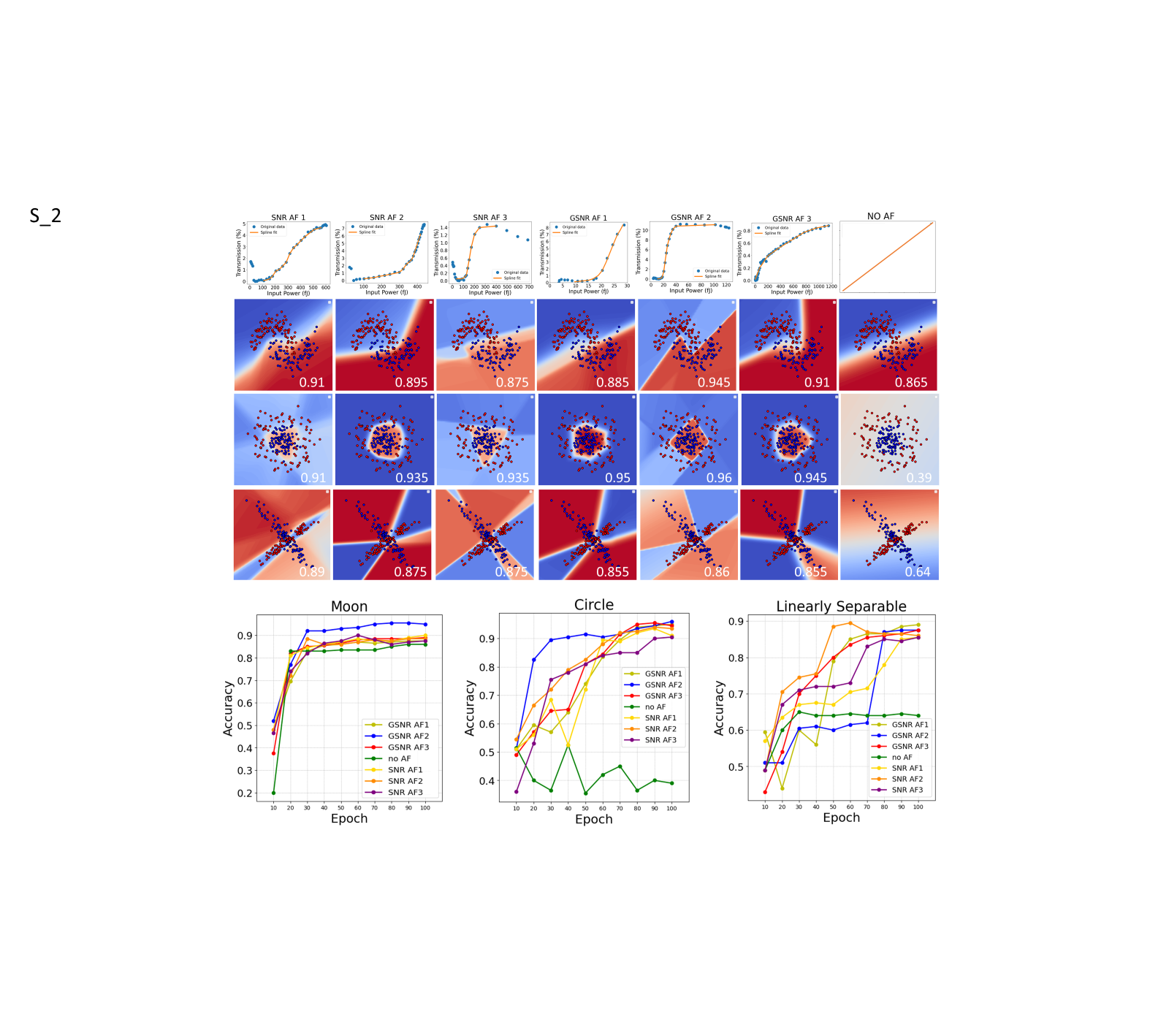


**Fig. S9 | Performance of NAFs on Three Binary Classification Datasets**

For the two classic image datasets, MNIST and CIFAR-10, we compared the performances of the models using graphene-silicon integrated ANA nonlinear response activation functions (GSNR AFs). MNIST is a classic dataset used for validating the performance of optical neural networks (ONNs). As shown in Fig. S10, all the activation functions demonstrate commendable performance on the MNIST dataset. Interestingly, the models also perform well without any activation functions. This is attributed to the strong linear separability of the MNIST dataset, allowing for the recognition and classification of numeric features even in the absence of activation functions. Therefore, to further investigate the performance of optical activation functions, it is essential to test their performance on more complex datasets that are closer to real application challenges.


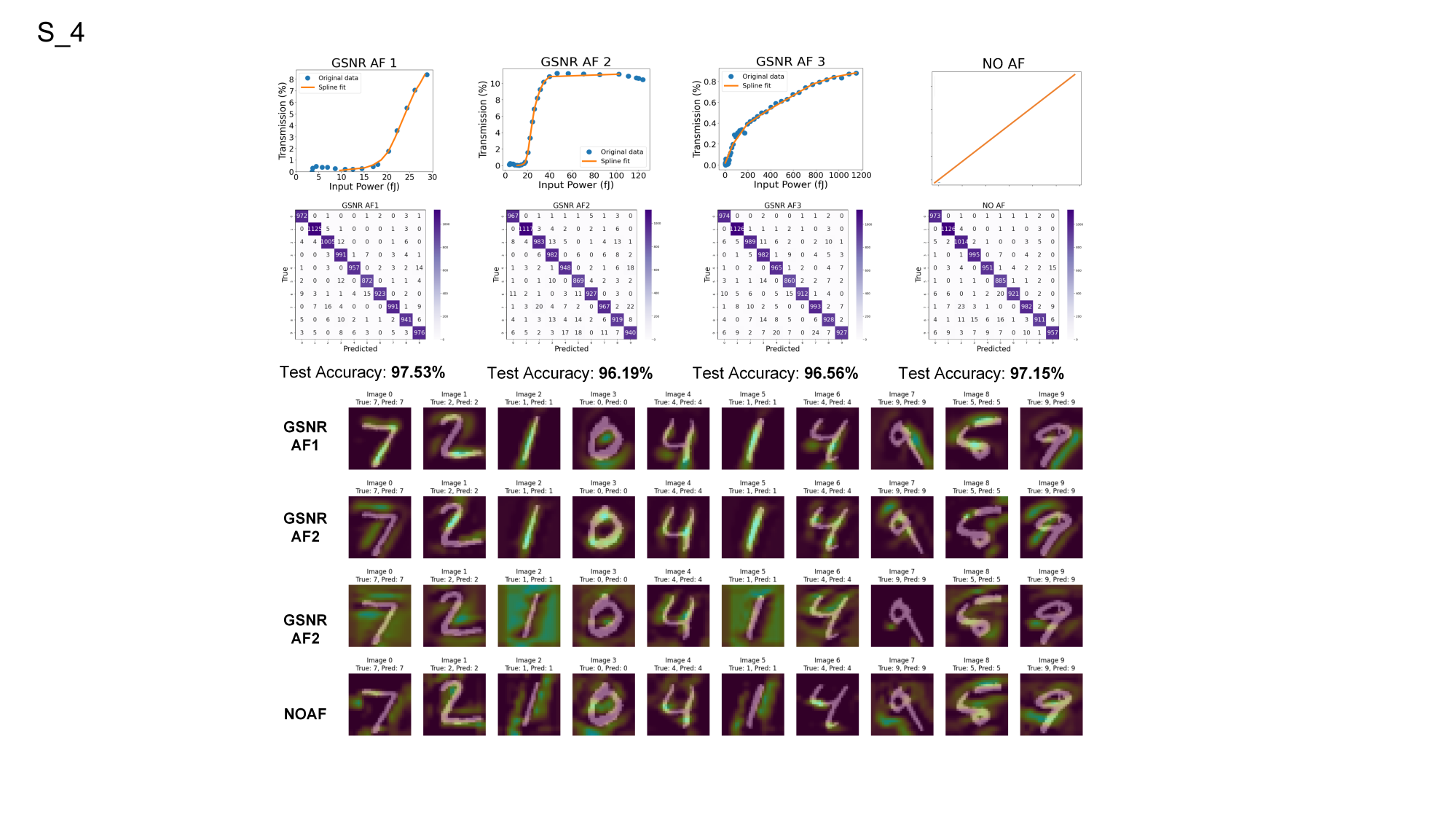


**Fig. S10 | Performance of OAFs on MNIST**

Consequently, we trained and tested models incorporating optical activation functions on the CIFAR-10 dataset. The CIFAR-10 dataset provides more complex images with higher background noise, demanding more robust feature extraction capabilities from the model. As illustrated in Fig. S11, the model employing the GSNR AF1 activation function exhibits significantly superior feature extraction capabilities compared with models integrating other activation functions, enabling the identification of more detailed and accurate features. The test accuracy reached 83%, even surpassing a series of traditional mathematical activation functions (such as tanh, sigmoid, etc.).


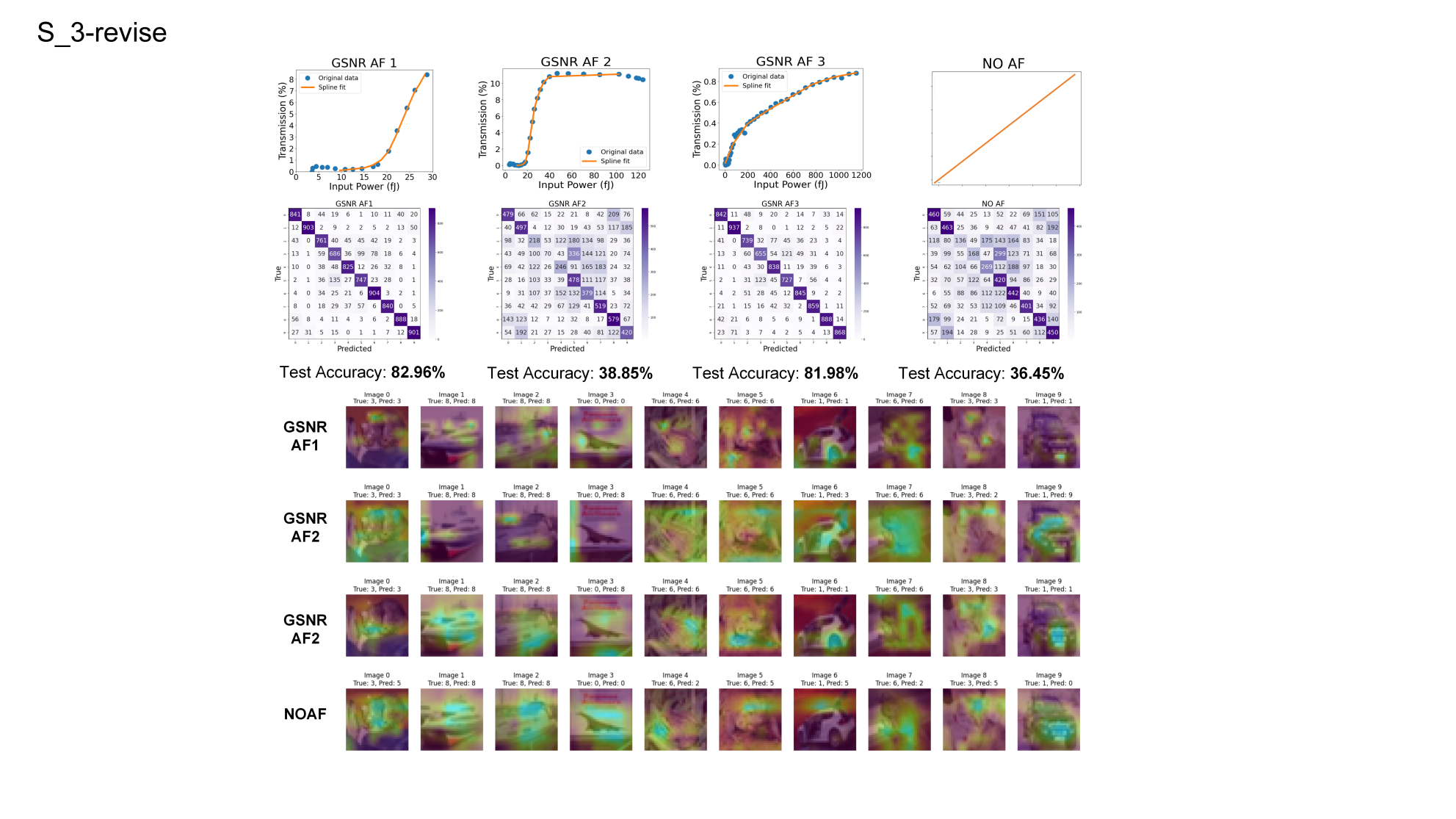


**Fig. S11 | Performance of OAFs on CIFAR-10**

In addition, to verify the effectiveness of the activation function we proposed compared with common activation functions, We conducted experimental simulations, as Fig. S12 shows. We compared graphene silicon/nonlinear heterojunction response activation functions (GSNR AF1, the yellow line, GSNR AF2, the blue line and GSNR AF3, the red line) with ReLU (the purple line), tanh (the orange line), and sigmoid (the pink line) by training them in the GSNR AFs training set, and used the case without an activation function (the green line) as a reference.

From the results, it can be seen that the effect of the activation function we proposed in the GSNR AF1 and GSNR AF2 training set have significant performance advantage over the common tanh and sigmoid activation functions and is comparable to the performance of ReLU.


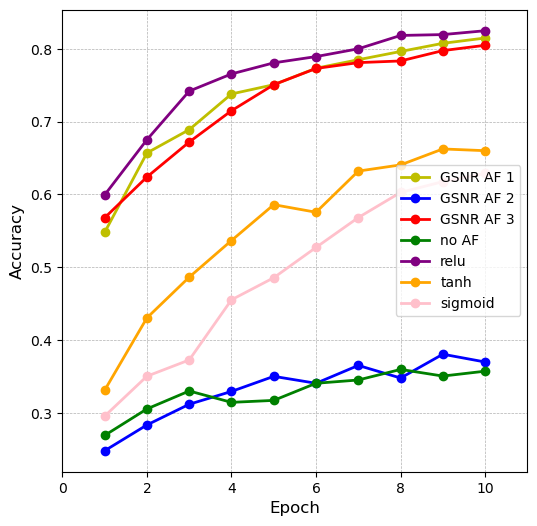


**Figure S12 | The comparison of the training performance between the activation function proposed in this work and common activation functions. Among them, the yellow line represents the GSNR AF1 generated by ANA, the red line is the result of ReLU, the blue line is the result of tanh, and the green line is the result of sigmoid trained in the GSNR AF1 training set, with the case of no activation function (the purple line) used as a reference.**

Here, we supplement the training of the activation function presented in Figure 3e of the manuscript on the CIFAR - 10 dataset. The supplementary results of the GSNR activation function yield a classification accuracy of 81.77% on the CIFAR - 10 dataset.
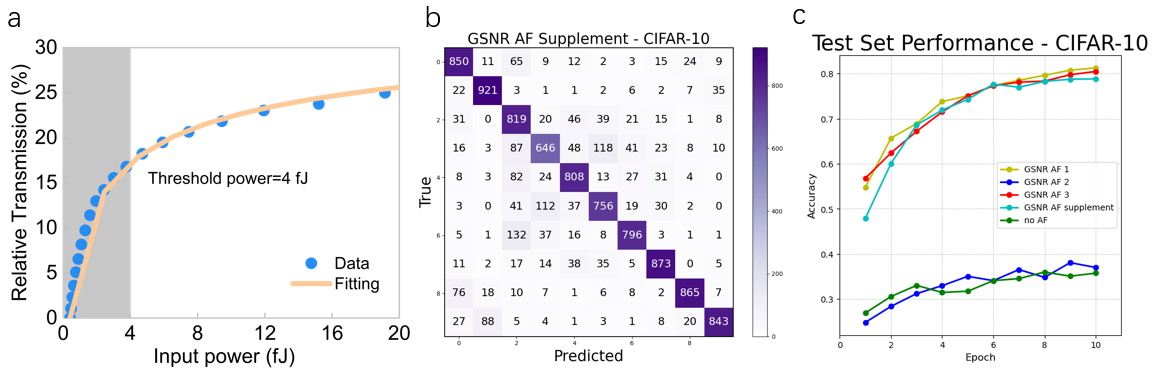


**Figure S13 | a.** Figure 3e of manuscript, which shows the saturable absorption curve of the PhC cavity with graphene, with a threshold power of 4 fJ (50% saturation transmission rate). It is generated by a broadband pulse without filtering. **b.** Confusion matrix using supplement GSNR AF on the CIFAR-10 dataset. **c.** Validation classification accuracy results using different activation functions on CIFAR-10 dataset.

**Section Ⅹ - Prospective Performance Evaluation of the On-Chip Picosecond-Pulsed Optical Neural Network (Based on the performance of ANA)**

In this section, based on the performance of the all-optical nonlinear activator, we project the ultimate performance of our optical neural network architecture under ideal conditions. In the architecture proposed in this paper, a single neuron consists of two inverse-designed wavelength division multiplexers, sixteen linear computing units made of phase-change materials, and one nonlinear activator.

Among them:The inverse-designed wavelength division multiplexer has the potential to reach a size of 25×7.5 µm² through future continuous optimization. As for the linear computing module, due to the utilization of the relatively large refractive index variation range of the phase-change material, we only need to cover it on a 5-μm-long waveguide to achieve its function. Finally, the ANA we proposed can realize the nonlinear activation function at a size of 15×5 µm². Therefore, based on the structural diagram shown in Figure S14, we optimistically estimate the area of a single neuron to be 25×30 µm².


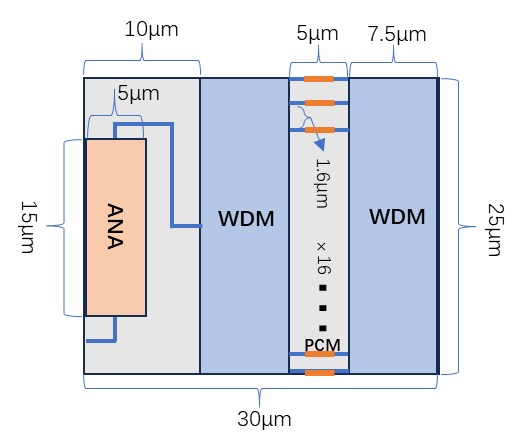


**FigureS14 | Structural diagram of proposed single neuron.**

Thus, a single optical neuron can perform 16 weight operations and one nonlinear activation. Under the assumption that our picosecond pulsed optical neural network has a 100 GHz clock rate and 16 wavelengths, it can operate on the signal at a speed of 1.6 TOPS. Consequently, the computing power density of our single optical neuron can be given as (30×25 μm^2^)^-1^×1.6 TOPS, which equals 2.13×10^3^ TOPS·(mm^2^)^-1^. Note that this calculation only considers a single neuron, while the actual multi-layer cascaded MMI and waveguides in the network would reduce the practical computing density.

Furthermore, leveraging the near-zero power consumption characteristic of nonvolatile phase-change materials in linear weighting operations, and assuming negligible insertion losses, the system's energy consumption is predominantly determined by the all-optical nonlinear activator (ANA). Through optimization of device insertion loss and resonator Q-factor, the ANA can potentially achieve an ultralow threshold power of 30 fJ (as demonstrated by our graphene-silicon ANA's sigmoid activation function at 1540 nm). Given that each neuron performs 16 multiply-accumulate operations, this translates to merely 1.875 fJ per operation. Consequently, each neuron requires at minimum a 30 fJ energy pulse as the activation pump light and an additional 30 fJ optical pulse as the signal to be activated and transmitted to the next neuron layer. At a 100 GHz clock rate, the power consumption per neuron calculates to 30 fJ × 100 GHz, equating to 3 mW. Under ideal conditions, this enables a single neuron to achieve an ultimate computational energy efficiency density of 0.71×10⁶ TOPS/W/mm². We have compared these results with state-of-the-art electronic GPUs and other optical computing architectures in Table S2, demonstrating the significant potential of our architecture and activator in terms of computational density and power efficiency.

**Table S2: Comparison of different state-of-the-art ONN architectures and electronic GPUs in terms of energy, area efficiency, and power efficiency.**

| Device | Energy consumption per operation (fJ) | Computing power density  (TOPs·mm^-2^) | Computing power energy efficiency density (TOPsW^-1^mm^-2^) |
| --- | --- | --- | --- |
| This work | 1.875 | 2.13×10^3^ | 0.71×10^6^ |
| *Nature.* **606**, 501–506, 2022^8^ | 3.45×10^2^ | 3.5 | 6.09×10^2^ |
| *Nature.* **589**, 52–58, 2021^9^ | 8.5 | 1.2 | 1.18×10^4^ |
| *Science* **384**, 202–209, 2024^10^ | 6.2 | 8.8×10^2^ | 1.26×10^4^ |
| NVIDIA GB200^11^ | 4.69 | 3.54×10^2^ | 1.31×10^-1^ |
| NVIDIA H100^12^ | 22.1 | 38.9 | 5.56×10^-2^ |
| AMD MI 300X^13^ | 35.9 | 8.79 | 1.17×10^-2^ |
| Intel Gaudi 3^14^ | 30.7 | 17.8 | 1.97×10^-2^ |

Moreover, we estimated the area required to achieve recognition of the MNIST dataset via the picosecond pulsed optical neural network. As shown in Fig. S15, light is first split into 16 beams through the WDM, then modulated with weight by phase-change materials, and finally converged and output by the reverse WDM. Therefore, within one-time unit, a device can perform 16 multiplication operations. Thus, we need to calculate only the number of multiplication operations required for the entire CNN process to estimate the required number and area for the ONN. Here, we did not implement the fully connected part and only calculated the resources required for the convolutional part.

The structure of the CNN is shown in Fig. S15, and we sequentially calculate the number of multiplication operations required for each convolutional layer:

1. Convolutional layer 1 CONV_1:

Input size: 28 × 28 × 1

Convolutional kernel: 3×3

Output channel: 4

Multiplication times=28 × 28 × 1 × 3 × 3 × 4 = 28224

2. Convolutional layer 2 CONV_2:

Input size: 14 × 14 × 4

Convolutional kernel: 3×3

Output channel: 8

Multiplication times=14 × 14 × 4 × 3 × 3 × 8=56448

3. Fully connected layer FC

Input size: 7 × 7 × 8

Output channel: 10

Multiplication times=7 × 7 × 8 × 10=3920

4. Total multiplication times for all layers

CONV_1+CONV_2+FC = 28224+56448+3920 = 88592

5. The required number of photon devices can be obtained by multiplying the total number of times by 16:

88592/16=5537

6. Multiplying the required area of the photon device yields the required area of the CNN:

5537 × 30 μm × 25 μm ≈ 4.15 mm^2^


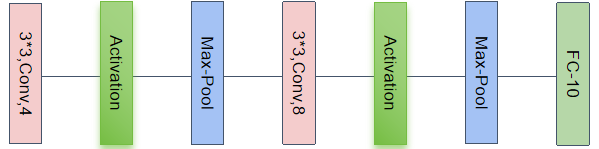


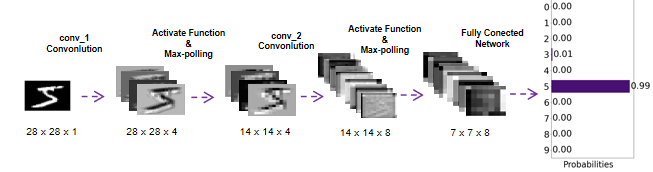


**Fig. S15 | The structure of ONN architecture.**

**References**

1. Hu, J. Planar chalcogenide glass materials and devices. (Massachusetts Institute of Technology, 2009).

2. Joannopoulos, J. D., Johnson, S. G., Winn, J. N. & Meade, R. D. Photonic Crystals: Molding the Flow of Light - Second Edition. in (Princeton University Press, 2011). doi:10.2307/j.ctvcm4gz9.

3. Hu, J. Ultra-sensitive chemical vapor detection using micro-cavity photothermal spectroscopy. *Opt. Express, OE* **18**, 22174–22186 (2010).

4. Lin, H. *et al.* Chalcogenide glass-on-graphene photonics. *Nature Photon* **11**, 798–805 (2017).

5. Boyd, R. W. Chapter 2 - Wave-Equation Description of Nonlinear Optical Interactions. in *Nonlinear Optics (Third Edition)* (ed. Boyd, R. W.) 69–133 (Academic Press, Burlington, 2008). doi:10.1016/B978-0-12-369470-6.00002-2.

6. Robinson, J. T., Preston, K., Painter, O. & Lipson, M. First-principle derivation of gain in high-index-contrast waveguides. *Opt Express* **16**, 16659–16669 (2008).

7. Ullah, S. *et al.* Graphene transfer methods: A review. *Nano Res.* **14**, 3756–3772 (2021).

8. Ashtiani, F., Geers, A. J. & Aflatouni, F. An on-chip photonic deep neural network for image classification. *Nature* **606**, 501–506 (2022).

9. Feldmann, J. *et al.* Parallel convolutional processing using an integrated photonic tensor core. *Nature* **589**, 52–58 (2021).

10. Xu, Z. *et al.* Large-scale photonic chiplet Taichi empowers 160-TOPS/W artificial general intelligence. *Science* **384**, 202–209 (2024).

11. NVIDIA DGX B200. *NVIDIA* https://www.nvidia.com/en-us/data-center/dgx-b200/.

12. NVIDIA H100 Tensor Core GPU Datasheet. *NVIDIA* https://resources.nvidia.com/en-us-tensor-core/nvidia-tensor-core-gpu-datasheet.

13. AMD Instinct^TM^ MI300X Accelerators. *AMD* https://www.amd.com/en/products/accelerators/instinct/mi300/mi300x.html.

14. Intel® Gaudi® 3 AI Accelerator White Paper. *Intel* https://www.intel.com/content/www/us/en/content-details/817486/intel-gaudi-3-ai-accelerator-white-paper.html.
